# Crystal structures of ternary complexes of archaeal B-family DNA polymerases

**DOI:** 10.1371/journal.pone.0188005

**Published:** 2017-12-06

**Authors:** Heike M. Kropp, Karin Betz, Johannes Wirth, Kay Diederichs, Andreas Marx

**Affiliations:** Konstanz Research School Chemical Biology, University of Konstanz, Baden-Württemberg, Konstanz, Germany; New England Biolabs Inc, UNITED STATES

## Abstract

Archaeal B-family polymerases drive biotechnology by accepting a wide substrate range of chemically modified nucleotides. By now no structural data for archaeal B-family DNA polymerases in a closed, ternary complex are available, which would be the basis for developing next generation nucleotides. We present the ternary crystal structures of KOD and 9°N DNA polymerases complexed with DNA and the incoming dATP. The structures reveal a third metal ion in the active site, which was so far only observed for the eukaryotic B-family DNA polymerase δ and no other B-family DNA polymerase. The structures reveal a wide inner channel and numerous interactions with the template strand that provide space for modifications within the enzyme and may account for the high processivity, respectively. The crystal structures provide insights into the superiority over other DNA polymerases concerning the acceptance of modified nucleotides.

## Introduction

DNA replication is the key process in all living cells. Even though the composition of the replication machineries may differ between archaea, eukaryotes and bacteria, all use DNA dependent DNA polymerases (DNA pols) for the replication of the genome. As DNA polymerases not only accurately duplicate DNA but also show the ability to accept chemically modified nucleotides, these enzymes are widely used in biotechnological applications like next-generation sequencing (NGS) [1–6], nucleic acids diagnostics [7–9], DNA conjugation [10–13] and SELEX [14, 15]. In various studies, it was reported that the archaeal B-family DNA polymerases accept a broader range of modified nucleotide triphosphates as substrate than the bacterial A-family DNA pols. [16, 17] Thus, B-family DNA pols are the enzymes of choice for important biotechnological applications like NGS sequencing.[1]

So far, only structural data of viral (RB69 [18, 19], Φ29 [20]), bacterial (E. coli DNA pol II[21]) and eukaryotic (yeast DNA pol α [22], δ [23] and ε [24]) B-family DNA pols complexed with primer, template and incoming dNTP are available. Remarkably, these enzymes differ in several aspects like in the coordination of the active site metal ions that are essential for catalysis. Therefore, the available data does not allow to predict how archaeal DNA pols bind and coordinate the triphosphate and metal ions in a ternary state that is poised for catalysis. The only crystal structures of archaeal DNA pols are apo structures of enzymes from *Thermococcus kodakaraensis* (KOD [25]), *Thermococcus sp*. *9°N-7* (9°N [26]), *Thermococcus litoralis* (Deep Vent [27]), *Pyrococcus furiosus* (Pfu [28]), *Pyrobaculum calidifontis* (Pc [29]) and *Thermococcus gorgonarius (*TGO [30]) as well as the binary structures of KOD [31], 9°N [31] and Pfu [28] DNA pols. Due to a missing ternary crystal structure, the viral RB69 was used as a general model for B-family DNA pols [27, 28] even though the sequence identity with archaeal polymerases e.g. 18.1% between KOD and RB69 DNA pols, is low.

We report the first crystal structures of archaeal B-family DNA pols (KOD and 9°N) complexed with primer, template and incoming dNTP in the active site in a state just prior to catalysis. The study provides insights into the active complex of archaeal DNA polymerases for the first time. This might be important for the development of next generation nucleotides for future biotechnological applications. Furthermore, the obtained data elucidates similarities and differences with other B-family DNA pols like DNA pols δ and RB69, which adds to the understanding of the evolutionary relationship within this enzyme family.

## Material and methods

### Protein purification

#### KOD DNA pol

For the crystallization experiments the exonuclease deficient mutant D141A, E143A was used. KOD DNA pol was cloned into a pET24a vector and overexpressed in E.coli BL21(DE3) using a codon-optimized sequence as described by Bergen *et*. *al*. [31] 2 L of LB-Media containing 34 mg/L Kanamycine, were inoculated with each 10 mL overnight culture. The expression was induced at an OD_600_ of 0.6 using 1 mM IPTG. Cells were harvested (4000 rpm and 4°C) at an OD_600_ of 2.0. The cell pellet was resuspended in approx. 30 mL low salt buffer A (10 mM Tris-HCl pH 8.0, 1 mM DTT, 0.1 mM EDTA, 200 mM NaCl, 10% Glycerol) and lysed for 1 h at 37°C by adding lysozyme to a final concentration of 1 mg/mL and PMSF to a final concentration of 1 mM. The lysate was heat denaturated at 75°C for 20 min and the cell debris were pelleted by ultracentrifugation (24 000 rpm, 4°C, 1 h). The genomic DNA was precipitated using a 5% w/v polyethylenimine solution that was added dropwise to the supernatant (approx. 400 μL per 40 mL lysate). The supernatant was incubated for 20 min on ice, followed by pelleting of the DNA (4000 rpm, 20 min). The supernatant was applied to a 5 mL Hi-Trap^TM^ Heparin HP column (GE Healthcare) with the low salt buffer and the proteins were eluted by increasing concentrations of NaCl using the high salt buffer B (10 mM Tris-HCl pH 7.0, 1 mM DTT, 0.1 mM EDTA, 1 M NaCl, 10% Glycerol) and a step wise gradient (0% B 10 mL, 0–5% 50 mL, 5% 50 mL, 5–10% 50 mL, 10% 50 mL, 10–20% 300 mL, 20–100% 200 mL at 1 mL/min). Purest fractions, eluting between 10–20% buffer B (as determined by SDS-PAGE) were pooled and concentrated with repeated resuspending in 10 min intervals at 4000 rpm and 4°C using a 50,000 kDa Vivaspin (Sartorius) to a final volume of 1 mL. The protein was further purified in four portions à 250 μL by gel filtration chromatography using a Superdex 75 16/600 column (GE Healtcare) and the storage buffer (10 mM Tris-HCl pH 7.8, 1 mM DTT, 0.1 mM EDTA, 200 mM NaCl, 10% Glycerol). The purest fractions (as determined by SDS-PAGE) were pooled and concentrated using a 50,000 kDa Vivaspin (Sartorius) to a final concentration of 15–20 mg/mL as determined by Bradford-assay and stored at 4°C.

#### 9°N DNA pol

The exonuclease deficient 9°N DNA polymerase (D141A, E143A) [32] was purified as described by Bergen *et*. *al*. [31]

### Activity assay

The enzyme’s polymerase activity was determined by amplifying a 1044 bp long part of the KOD DNA pol gene using 1x Phusion HF Buffer (New England Biolabs), 0.2 ng/μL template (pET24a vector with the KOD DNA pol gene), 500 nM forward primer (5’-d(TTTGCACTGGGTCGTGATG)), 500 nM reverse primer (5’-d(CAGTTCCAGTGCACCCGGC)), 200 μM dNTPs, DNA pol (5 nM KOD DNA pol, 5 nM 9°N DNA pol or 2 units Phusion HF (New England Biolabs, 2000 units/mL)) and 28 μL H_2_O (S1 Fig). PCR program: 95°C 5 min; 39 repeats of: 95°C 30 sec, 55°C 30 sec, 72°C 60 sec; 72°C 10 min.

### Protein crystallography

Primer and template were purchased HPLC purified from Biomers. 2´3´-dideoxy-cytidine 5´-triphosphate (ddCTP) and 2’-deoxy-adenosine-5’-triphosphate (dATP) were purchased from Jena Bioscience.

#### KOD DNA pol

3.80 μL of the primer (5’- d(GAC CAC GGC CA), 6 mM) and 3.80 μL of the template (5’-d(AAC TGT GGC CGT GGT C), 6 mM) strands were heated to 95°C and stepwise cooled to 4°C over 30 min. 1.40 μL MgCl_2_ (1M) and 1.40 μL MnCl_2_ (1 M) were added at a final concentration of 10 mM. For the enzymatic termination of the primer strand, 5.69 μL ddCTP (10 mM) was added followed by the protein, diluted with storage buffer, at a final concentration of 8.25 mg/mL giving a ratio of protein:DNA:ddCTP of 1:1.2:3. The solution was incubated at 55°C for 30 min, followed by the addition of 18.98 μL dATP (10 mM) in a protein:dATP ratio of 1:10. The solution was incubated at 30°C for 45 min and filtered through a 0.1 μm sterile filter (Ultrafree Centrifugal Filters, Millipore). Crystallization setups were done using the sitting drop vapor diffusion method with a Gryphon robot (ARI robots) and commercially available screens. The protein was mixed with the reservoir solution in ratios of 1:2, 1:1 and 2:1 giving a final drop size of 0.8–0.9 μL. Diffracting crystals grew in the H7 condition of the Morpheus MD1-46 screen (Molecular Dimensions) with each 0.2 M of the amino acids DL-glutamatic acid monohydrate, DL-alanine, glycine, DL-lysine monohydrochloride and DL-serine, 0.1 M sodium Hepes/ MOPS buffer pH 7.5 and 50% v/v of a mixture of glycerol (40%) and PEG 4000 (20%). Before freezing crystals were cryoprotected in the reservoir solution containing 20% ethylene glycol.

Diffracting crystals containing a closed ternary complex could be reproduced in the H7 condition as well as be grown in the A3, A7, C12 and E3 of the Morpheus MD1-46 Screen.

#### 9°N DNA pol

Ternary 9°N crystals were prepared similar to the KOD crystals. The protein was mixed with the annealed p/t duplex (same sequence as for KOD crystals), ddCTP and dATP in a ratio of 1:1.2:3:10. MgCl_2_ and MnCl_2_ were added to a final concentration of 10mM each. After addition of ddCTP and dATP the mixture was incubated for 30min at 30°C each. The final protein concentration was 8.3mg/ml. After filtration through a 0.1 μm sterile filter crystallization was performed. Well diffracting crystals grew in condition E10 of the PEG RX screen (Hampton Research) containing 6% Isopropanol, 0.1M sodium acetate trihydrate pH 4.5 and 26% PEG 550 MME. Before freezing crystals were cryoprotected in the reservoir solution containing 20% Glycerol. The crystals containing two or three divalent metal ions in the active site were harvested from the same crystallization drop.

#### Structure refinement

Data for the project was collected at the beamlines PXI and PXIII at the Swiss Light Source (SLS), Paul-Scherrer Institute, Villigen, Switzerland. Data reduction was performed with the XDS package [33] and structures were solved by molecular replacement with PHENIX using the binary KOD exo^-^ [31] and binary 9°N exo^-^ [31] structures. Refinement was performed with PHENIX [34] and model rebuilding was done with COOT.[35] In iterative rounds of refinement and model building geometry was validated using the MolProbity Server.[36, 37] In the KOD and 9°N models the C-terminal amino acids were not modelled due to missing electron density. Side chains with weak electron density were not deleted but modelled in a common rotamer conformation and high B-factors demonstrate their flexibility. The restraint file for the ligand DTP was generated using the Grade webserver.[38] Figures were generated with PyMOL.[39] The figures showing the inner channels of the DNA pols (S9 Fig) were generated using Chimera.[40] For comparison of enzymes, the complete complexes were superposed in PyMOL. To determine rmsd values only Cα atoms were superposed in PyMOL with the default values (e.g. outlier rejection cutoff in RMS = 2, outlier rejection cycles = 5). Data collection and refinement statistics are summarized in S1 Table.

## Results

### Approach to obtain crystals of ternary complexes of DNA pol, primer/template, and dNTP

In order to obtain a closed, ternary structure of KOD and 9°N DNA pols, the approach used for generating the binary complex [31] was partially changed. First, to minimize the impurity of the DNA pols by truncated enzyme, we adopted the gradient of the cation-exchange chromatography run used for KOD DNA pol and decreased the volume per size-exclusion chromatography run from 1 mL to 250 μL for KOD and 9°N DNA pols. Second, the DNA sequence used for crystallization was changed and the 5’-Cy5 dye of the template was not used, to avoid any kind of perturbation by the dye. Third, the primer was terminated by the addition of ddCTP to the protein-DNA solution, to allow the later binding of a natural dATP in the active site [41, 42]. Here, the protein-DNA and the ddCTP/ dATP were incubated with 10 mM MgCl_2_ and 10 mM MnCl_2_ at elevated temperature and time to insure a full incorporation of the ddCTP as well as a closure of the enzyme. We used MgCl_2_ and MnCl_2_ simultaneously, as MgCl_2_ only did not lead to the crystallization of ternary complexes.

### Crystal structure of KOD DNA polymerase

**Fig 1:**
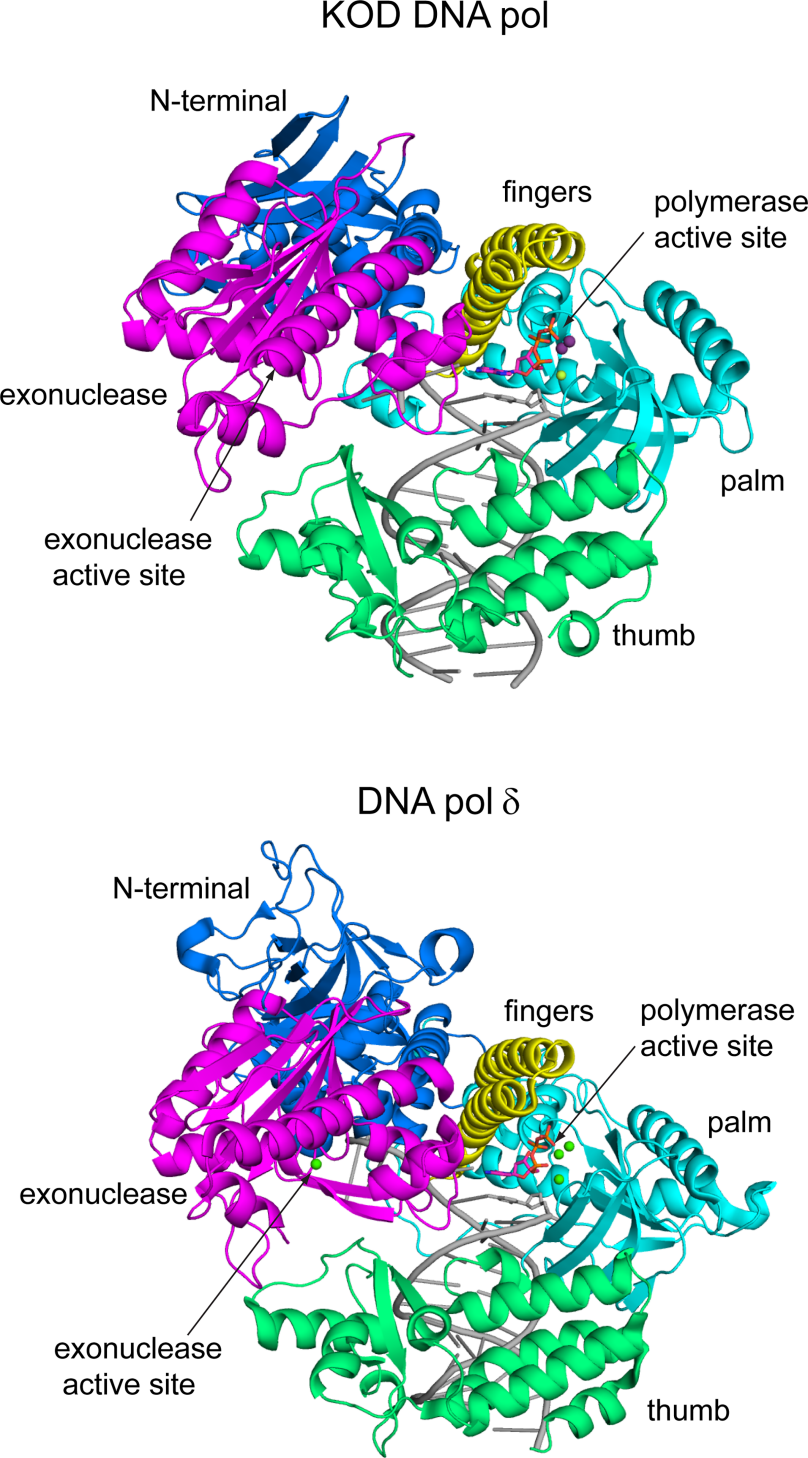
Comparison of the B-family DNA pols KOD and δ. The domains are color coded as followed: the N-terminal (blue), exonuclease (magenta), finger (yellow), palm (cyan) and thumb (green) domain. The p/t complex is shown in grey. The polymerase active site and the exonuclease active site are indicated by arrows. The bound dNTP as well as the metal ions are shown as sticks and spheres, respectively.

We crystallized both, KOD DNA pol and 9°N DNA pol in a closed ternary complex. The structures were solved using molecular replacement and the binary KOD exo^-^ [31] and binary 9°N exo^-^ [31] DNA pol structures as models, respectively. As both DNA pols are similar in sequence (sequence identity 27.4%) and structure (RMSD of 0.689 over 599 Cα atoms) we describe the KOD DNA pol structure in the following and only mention the 9°N DNA pol structure in cases of significant differences. The domain organization of KOD DNA pol is similar to DNA pol δ [23] (overall RMSD = 1.712 over 516 Cα atoms), showing interactions of the primer strand with the palm, thumb, finger and exonuclease domain (Fig 1).

**Fig 2:**
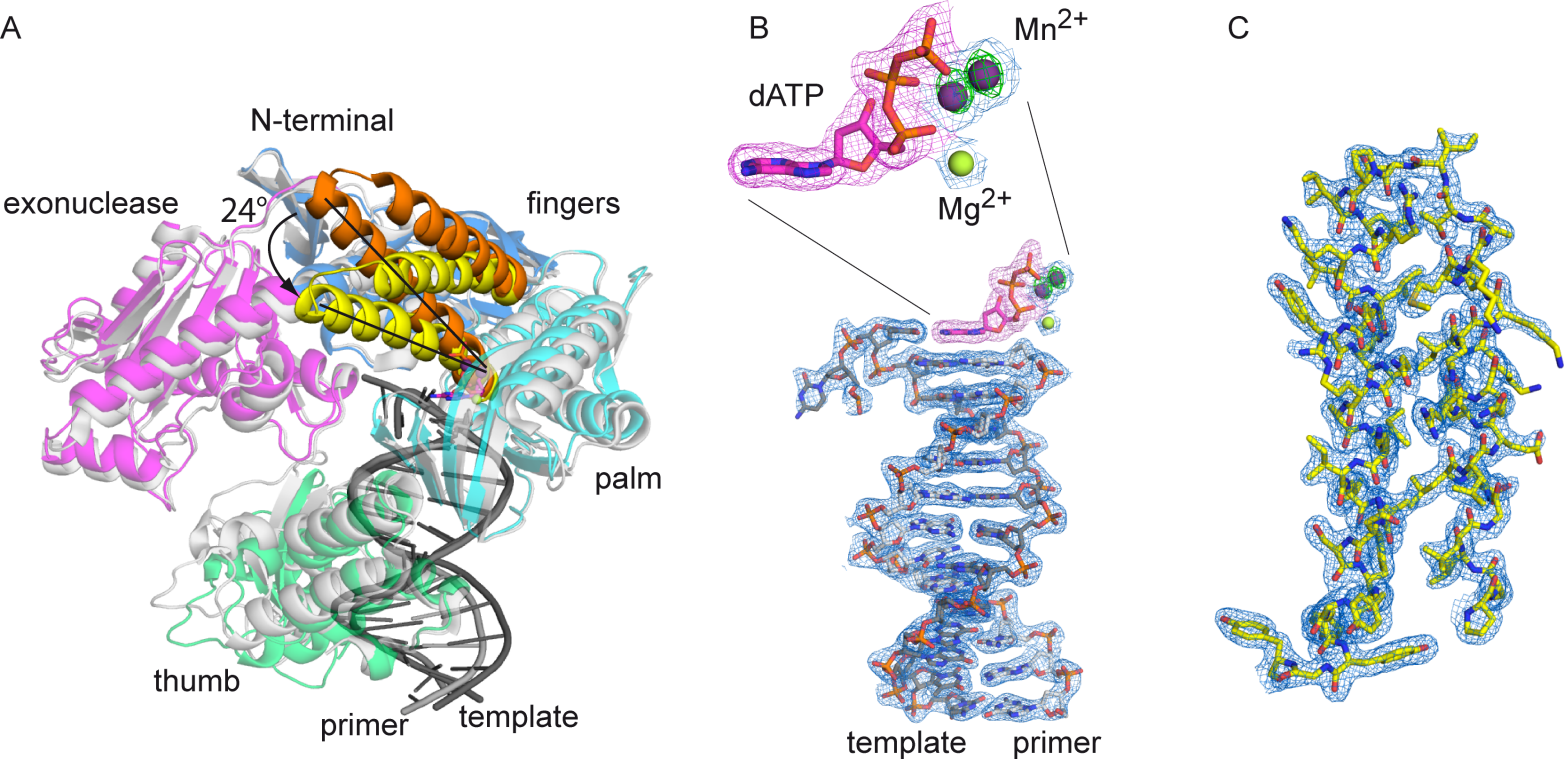
Overview of the closed ternary complex of KOD pol. (A) The ternary complex is color coded as seen in Fig 1, the finger domain of the ternary complex (yellow) is closed by approximately 24° compared to the finger domain (orange) of the superimposed binary KOD complex (grey). (B) The electron density of the primer/template is shown at 1 σ as a blue mesh, the omit map at 3 σ of the dATP is shown as a pink mesh, the electron density of the Mg^2+^ and the two Mn^2+^ ions is shown as a blue mesh at 1 σ with the anomalous signal at 3 σ as a green mesh for the two Mn^2+^ ions. (C) The electron density for the finger domain at 1 σ is shown as a blue mesh.

The palm domain of KOD DNA pol is structurally highly related to DNA pol δ’s palm domain (RMSD of palm domain = 1.031 over 135 Cα atoms) with the same structural elements of two long and one short α-helix as well as a six-stranded β-sheet (Fig 1) [23]. The N-terminal domain interacts with the last resolved nucleotide of the single stranded template. The finger domain of KOD DNA pol is structurally related to the finger domain of DNA pol δ, both showing two α helices which extend by approx. 40 Å, whereas RB69 DNA pol exhibits a longer finger domain which extends by approx. 60 Å and includes an additionally short α-helix [18]. In comparison to the binary structure of KOD DNA pol, the finger domain shows a closed conformation, indicated by a rotation of the two α-helices by approx. 24° (Fig 2A). Thereby, the amino acids interacting with the dATP move 2.8–6.6 Å (S2 Fig, measured from the Cα atoms), packing against the nascent base pair forming the active complex.

The DNA duplex in KOD DNA pol adopts B-conformation, with only the 3’-primer nucleotide having C3’-endo conformation [43], which is in contrast to A-family DNA pols like KlenTaq [44], T7 DNA pol [45] and Bacillus fragment (BF) DNA pol [46], where the DNA close to the active site adopts A-form.

The exonuclease domain of B-family DNA pols exhibits a β-hairpin, which is proposed to facilitate strand separation by keeping the template strand in place while the primer strand swings into the exonuclease active site, thus maintaining the DNA pol stably bound to the DNA template strand during execution of exonuclease activity. [47] For RB69 DNA pol it was shown that this β-hairpin plays a direct role in facilitating the movement of the primer strand to the exonuclease active site [47]. The structure of KOD DNA pol reveals that the β-hairpin is shorter than in RB69 DNA pol (12 and 18 amino acids, respectively) but has approximately the same length as observed for DNA pol δ (12 and 13 amino acids, respectively) (S3 Fig). However, the loop connecting the β-hairpin to the exonuclease domain is reduced in KOD DNA pol compared to DNA pol δ, leading to an overall shorter exonuclease domain. Thus, the β-hairpin in KOD DNA pol is located further away from the template strand, not undergoing interactions with the DNA.

KOD DNA pol has conserved sequence patches with DNA pol δ and RB69 DNA pol for the polymerase active site, the minor groove sensing residues, the palm residues interacting with the 5’ template and the triphosphate binding pocket (S4 Fig). Additionally, KOD DNA pol shares highly conserved sequence patches in the exonuclease, finger and N-terminal domain as well as the third metal binding site with DNA pol δ (S4 Fig).

### Active site coordination

**Fig 3:**
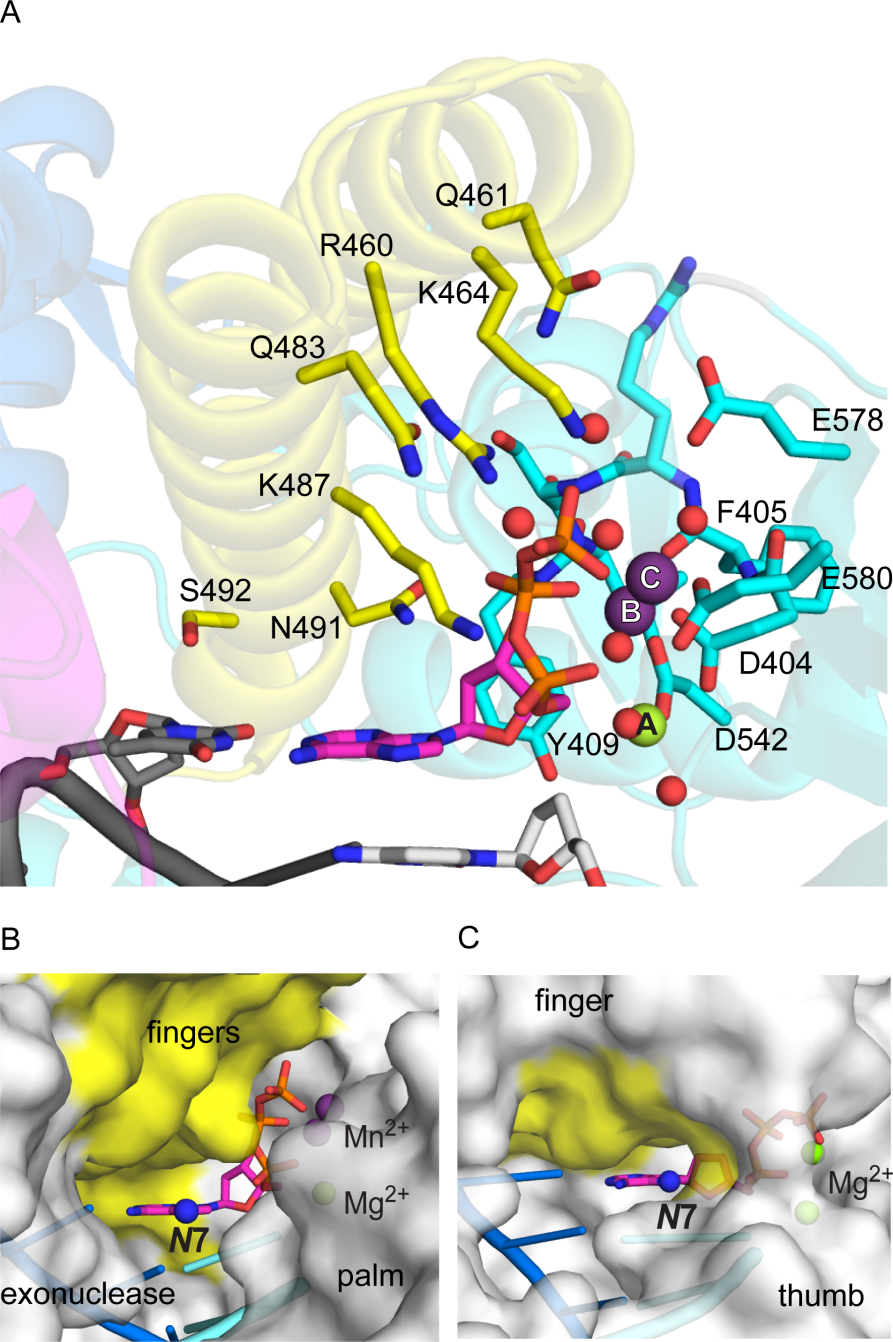
KOD DNA pol’s active site with bound dATP. (A) The metal ions are coordinated by residues of the palm domain (cyan). Metal ion A (Mg^2+^, green) is coordinated by two water molecules, D542, the α-phosphate of dATP (pink) and D404. Metal ion B (Mn^2+^, purple) is coordinated by the α-, β- and γ- phosphate, D404, F405 and D542. Metal ion C (Mn^2+^, purple) is coordinated by the γ-phosphate, E580, F405, D404 and three water molecules, whereof one molecule is coordinated by E578, one by E580 and one by K464 (yellow) of the finger domain. The dATP makes further direct contacts with conserved residues of the finger domain (yellow), N491, K487 and R460 as well as water mediated contacts to Q483 and K464. Additionally, a water mediated interaction with the not conserved Q461 (yellow) can be formed. (B, C) DNA pols’ protein surface is shown in grey, the template in blue, the primer in bright blue, the bound adenosine triphosphate in pink with the *N*7 atom indicated as a blue sphere; (B) KOD DNA pol shows the *N*7 atom pointing towards a wide open crevice between the finger (yellow), palm and exonuclease domain; (C) KlenTaq DNA pol shows the *N*7 atom pointing towards the O-helix of the finger domain (yellow) and the thumb domain, the crevice overall being narrower compared to KOD DNA pol.

KOD DNA pol binds the incoming dATP in a pocket formed by the palm and closed finger domain. The dATP adopts C3’ endo conformation and packs with its sugar against Y409, where the C2’ atom is closest to the aromatic ring of Y409, which explains the discrimination against ribo nucleotides [48]. The purine-pyrimidine base-pair stacks against S492 and N491 (Fig 3A), which are conserved in DNA pol δ and RB69 DNA pol (S4 Fig). The dATP makes further contacts with the conserved amino acid side chains of N491, K487, R460 and water mediated to Q483 (Fig 3A) of the finger domain. Additionally, KOD DNA pol forms water mediated interactions with the dATP via Q461 and K464 (Fig 3A), which are not conserved among the B-family polymerases. All of these interactions form hydrogen bonds to phosphate groups, where N491 and K487 interact with the β-phosphate, R460, Q483, Q461 and K464 with the γ-phosphate and K487 additionally with the α-phosphate. N491 can also form a hydrogen bond to the 3’-OH group of dATP. This formed pocket closes tightly around the triphosphate of the dATP, however leaving the Hoogsteen side with the *N*7 atom accessible to the solution (Fig 3B).

The structure exhibits three metal ions at the active site (Mg^2+^ in position A, Mn^2+^ in position B and C; Fig 3), which are coordinated by the enzyme and the triphosphate. The presence of the two manganese ions was determined by the anomalous signal (Fig 2B). The metal ions in position A and B are observed in other DNA polymerases like KlenTaq [49], RB69 [19] and T7 [45], where metal ion A is assumed to lower the pKa of the 3’OH group for the nucleophilic attack and B to facilitate the pyrophosphate leaving [50]. Also the octahedral coordination of metal ion A and B (Fig 3) is analogous to the observed coordination in various other DNA polymerases [50]. The third metal ion in position C however, was so far only observed in DNA pol δ (S5 Fig). In KOD DNA pol, the third ion is coordinated by the γ-phosphate, D404, E580 and three water molecules, whereof one water molecule is coordinated by E580, one by K464 and one by E578 in an octahedral manner (Fig 3).

As these three metal ions were not only found in reproduced crystals of KOD DNA pol but also in different crystallization conditions (data not shown), we assumed that this feature may be characteristic for archeal DNA polymerases. In addition, we obtained a structure of 9°N DNA pol in complex with dATP and also three metal ions bound in the active site (S6 Fig). The three metal ions show the same coordination as observed for KOD DNA pol (S6B Fig), with Mg^2+^ occupying the third metal binding site in 9°N DNA pol. Additionally, we also obtained a closed structure of 9°N DNA pol with a bound dATP, a Mg^2+^ in position A and a Mn^2+^ in position B (S6C Fig), missing the third metal ion.

### Comparison of DNA interactions

**Fig 4:**
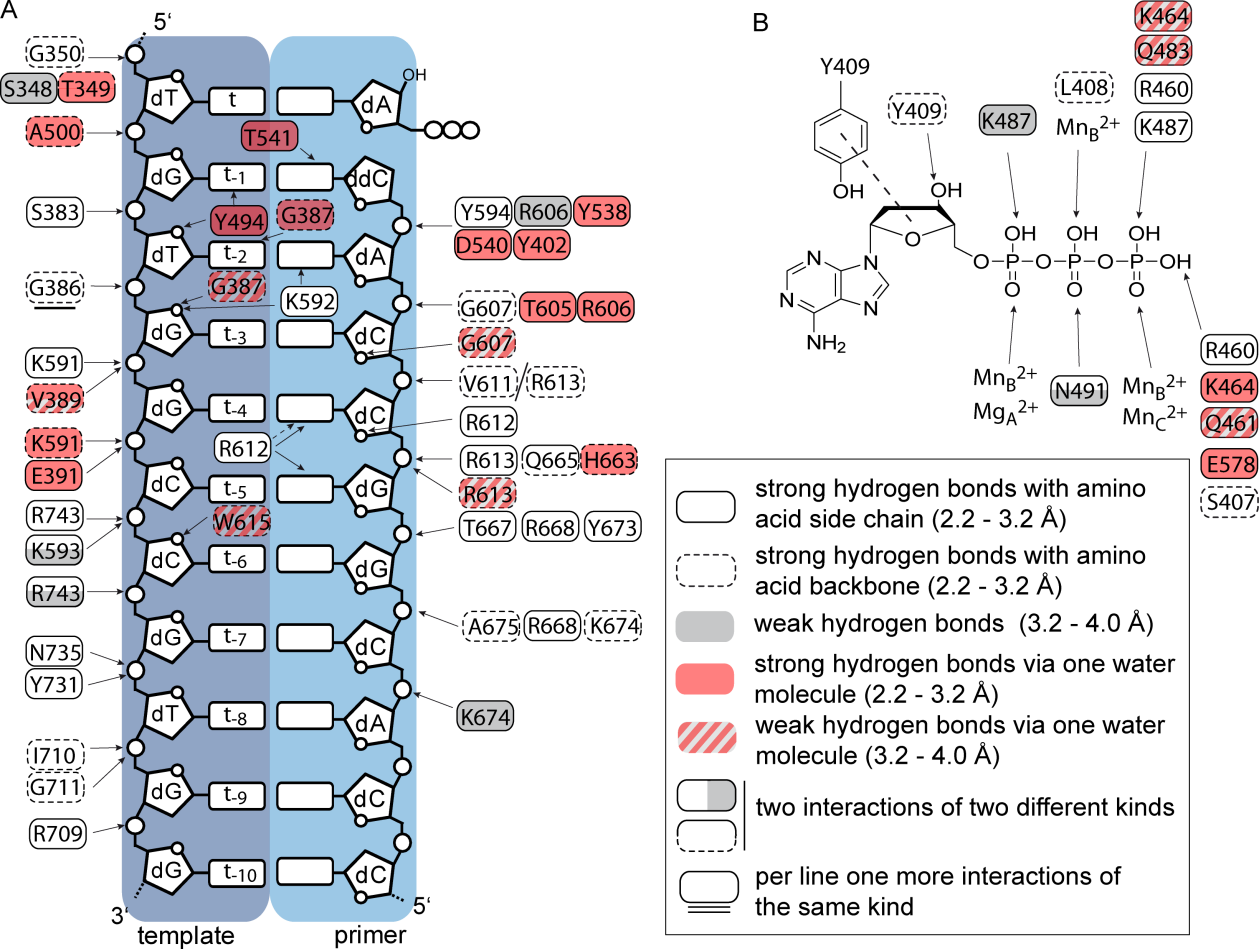
Interaction pattern of KOD DNA pol, according to the strength of the interaction. The interactions between the enzyme and the respective dNTP as well as between the enzyme and the template/ primer strand were assigned according to their strengths (see legend) (A) The interaction between the protein and the template and primer strand are shown. (B) The interactions with the dATP are shown, the stacking between Y409 and the sugar moiety is indicated by a dashed line.

KOD DNA pol accepts a wide range of modified nucleotides [12, 16, 17, 51–56] and shows high processivity among archeal DNA pols (> 300 bases) [57]. Interactions with the DNA substrate might be the causative. Thus, we assigned and compared the protein-DNA interactions for RB69, KlenTaq and KOD DNA pols and DNA pol δ (Fig 4 and S7 Fig). To account for the strength of the interaction, we separated the hydrogen bonds in direct strong bonds (2.2–3.2 Å, white) and direct weak bonds (> 3.2–4.0 Å, grey). Additionally, indirect hydrogen bonds which are formed via one water molecule were assigned (strong 2.2–3.2 Å and weak > 3.2–4.0 A).

Inspecting the interactions between the protein and the primer sugar-phosphate backbone, the DNA pols structures of RB69, KlenTaq and KOD DNA pols and DNA pol δ show no significant difference in the number and pattern of interactions. However, in case of the template sugar phosphate backbone, KlenTaq DNA pol (PDB ID: 3RTV) has various direct interactions with the 5’ template region (t to t-5) (S7 Fig). Here, KOD, RB69 DNA pols (PDB ID: 3NCI) and DNA pol δ (PDB ID: 3IAY) have fewer direct interactions. DNA pol δ and RB69 DNA pol form various water mediated hydrogen bonds (Fig 4 and S7 Fig). At the 3’ template region (t-6 to t-10) KlenTaq, RB69 and δ DNA pols undergo fewer direct interactions (4, 5 and 3, respectively) with the template backbone compared to KOD DNA pol, which has direct hydrogen bonds to every phosphate group. In total KOD DNA pol forms 10 direct hydrogen bonds to the 3’ template region (Fig 4).

In comparison to KlenTaq DNA pol and DNA pol δ, KOD and RB69 DNA pols form the fewest interactions to the nucleobases, with only 7 and 10 direct and water mediated hydrogen bonds, respectively. Both, KlenTaq DNA pol and DNA pol δ show about twice the interactions (21 and 17, respectively) with the nucleobases compared to KOD DNA pol. Out of these interactions, DNA pol δ and KlenTaq DNA pol form not only minor groove interactions but also protein interactions with the major groove of the bound DNA. In case of DNA pol δ, K444 of the β-hairpin and Q586 of the palm domain form the major groove interactions. Both interactions are not found in KOD DNA pol due to a shorter β-hairpin and a serine (S389) residue at position Q586 of DNA pol δ. For KlenTaq DNA pol, the interactions with the nascent major groove may proceed by R660, which vary depending on the nature of the incoming nucleotide.[49] Additionally, R587 was shown to change its conformation and positions itself in the major groove to form interactions with modified nucleotides [58], which could also apply for R677 pointing into the major groove at the template site.

The number of interactions with the oxygens of the sugar heterocycle moieties does not differ significantly between the four DNA pols.

Inspecting the binding of the triphosphates in the active sites of the four DNA pols (Fig 4B and S7 Fig), reveals overall a very similar binding pattern. All dNTPs have interactions with a lysine residue for the α- and γ-phosphate as well as with an arginine residue for the γ-phosphate. Noticeable, KOD DNA pol can form the most water mediated interactions with the γ-phosphate of the bound dATP (Fig 4B), whereas the other interactions are equal to RB69, KlenTaq and DNA pol δ. However, KlenTaq DNA pol is the only polymerase that has an interaction with the nucleobase (S7 Fig). [49] Thereby, KlenTaq DNA pol, also forms a very narrow pocket surrounding the dATP (Fig 3C), which leads to the positioning of the N7 atom close to the finger and thumb domain.

**Fig 5:**
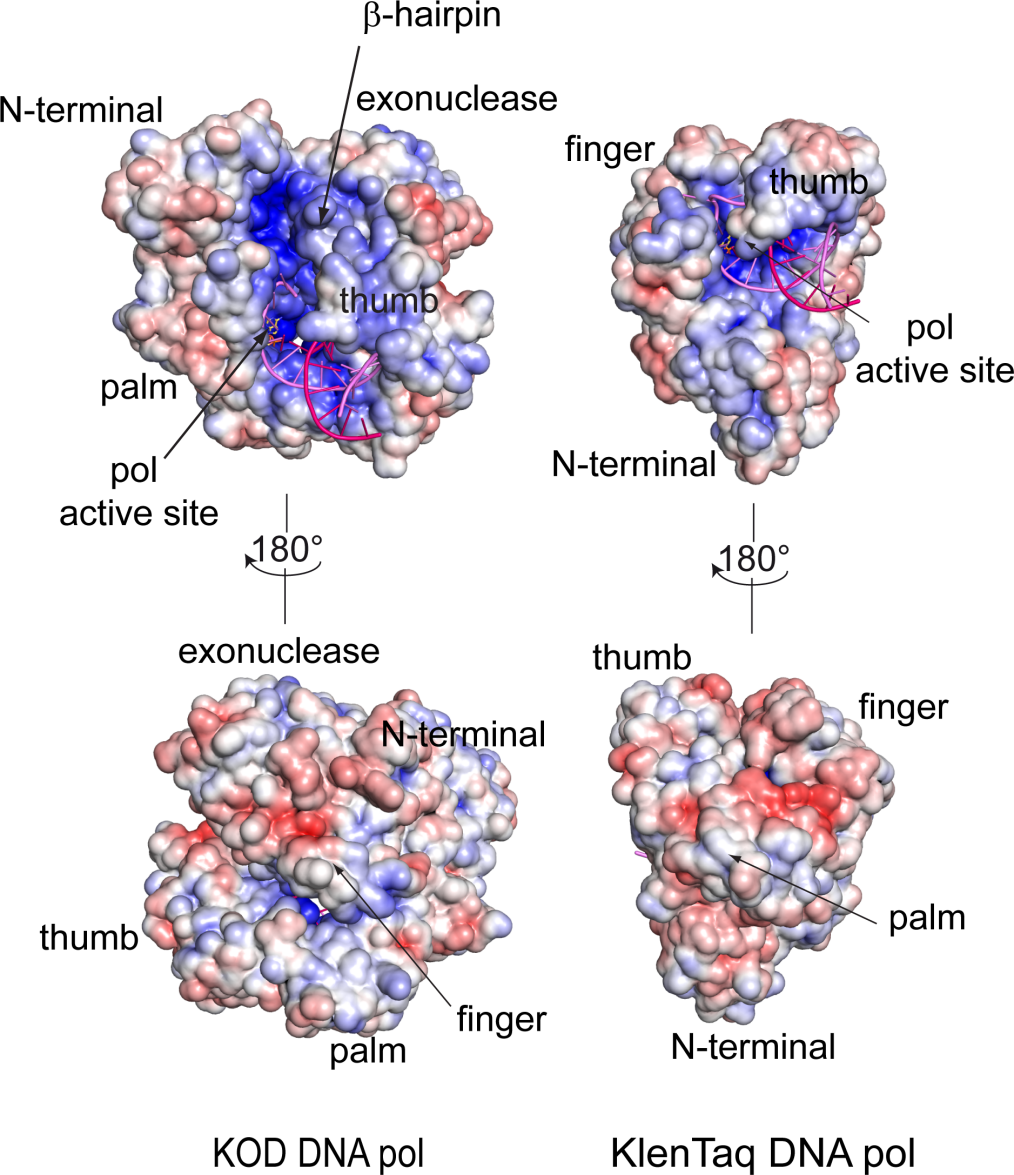
Electropotential map of KOD and KTQ DNA pols. The electropotential is shown from +6 (red) to -6 (blue) k_B_T/e (T = 310 K). The primer is shown in pink, the template in violet and the dNTP is shown as yellow sticks. KOD DNA pol exhibits a long crevice between the thumb and palm domain reaching up along the β-hairpin to the N-terminal domain, in which the single stranded template may bind. This crevice is missing in KlenTaq DNA pol, where the single stranded template leaves the polymerase between the thumb and finger domain.

In comparison to the A-family DNA pol KlenTaq, the B-family DNA pols KOD, RB69 and δ structures exhibit a long crevice with an electro positive potential, stretching from the DNA binding region at the thumb domain upwards along the β-hairpin and the palm domain, to the N-terminal domain (Fig 5 and S8 Fig). Due to the larger β-hairpin in DNA pol δ and DNA pol RB69, the formed crevice is narrower compared to KOD DNA pol. In the B-family DNA pols, this electro positive charged crevice between the β-hairpin and the N-terminal domain, could interact with the 5’ single stranded template overhang and thus further stabilize the melted DNA, which is not possible for KlenTaq DNA pol, due to the short and narrow DNA binding crevice. Additionally, in archaeal B-family DNA polymerases this crevice is supposed to bind the 5’ single stranded template for uracil recognition.[59, 60]

### Channel volume in the DNA pol structures

**Fig 6:**
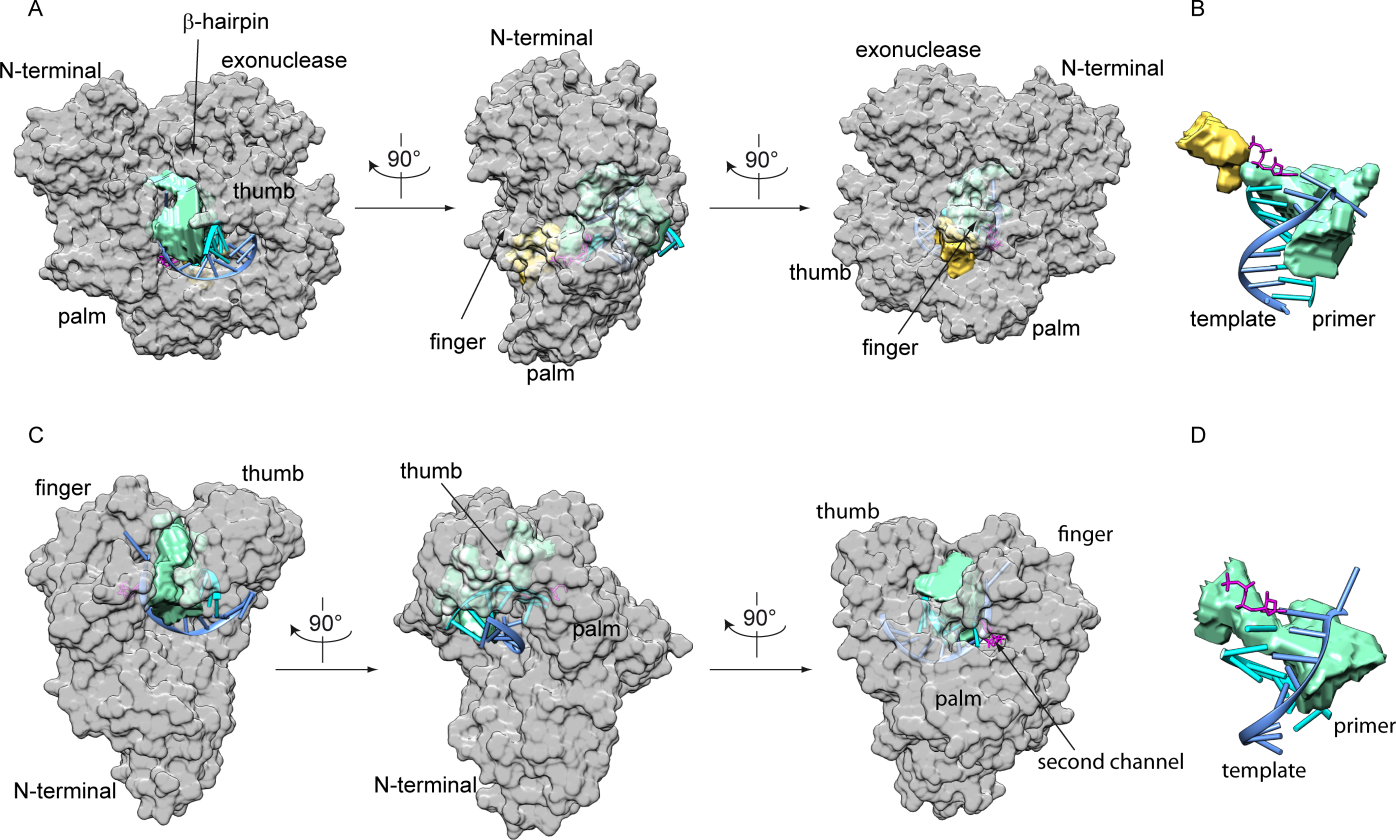
The channel volumes for KOD and KlenTaq DNA pols. The channel volumes were calculated with 3V algorithm for KOD (A and B) and KlenTaq (C and D) DNA pols, respectively. The protein is shown as grey surface, the primer in cyan and the template in blue. The bound dNTP is shown in magenta. A and C show the location within the enzyme, B and D the channels with respect to the DNA and triphosphate.

To estimate the channel volume that is accessible within the enzyme and that could be used for diffusion of the triphosphate and the leaving pyrophosphate as well as for modifications attached to nucleobases, we used the 3V algorithm [61]. The radii of the large and small sphere were set to 7.0 Å and 2.7 Å, respectively, as this turned out to be the radii best suited for the investigated DNA pols. KOD DNA pol has two channels within the enzyme (Fig 6), the larger one (green, 3939 A^3^) reaching from the β-hairpin and between the palm and the tip of the thumb domain along the DNA strand to the nucleobase of the triphosphate. The smaller channel (yellow, 1301 A^3^) spans from the phosphate groups of the bound triphosphate and between the finger and palm domain to the outside of the protein. These channels are also found in DNA pol δ with 2009 A^3^ and 2874 A^3^ as well as in RB69 DNA pol with 4354 A^3^ and 2898 A^3^, respectively (S9 Fig). For RB69 DNA pol the channel between the β-hairpin and N-terminal domain exhibits an “outer” channel (S9 Fig) which is not directly located at the DNA and is therefore hardly accessible for modifications. This “outer” channel occupies the electropositive crevice in which the single stranded template may bind, thereby enlarging the calculated channel volume.

In contrast, KlenTaq DNA pol has one major channel (3118 A^3^) stretching from the tip of the thumb domain along between the finger and thumb to the *O*-helix and the bound triphosphate (Fig 6). This channel is similar to the channel in the B-family DNA pols, reaching from the β-hairpin to the triphosphate. We were unable to calculate a second channel for KlenTaq DNA pol with the used radii. However, a narrow channel also exists in KlenTaq DNA pol, reaching from the triphosphate, underneath the *O*-helix to the palm domain (Fig 6C).

## Discussion

### Metal ion binding at the active site

The universal paradigm of DNA pols utilizing two metal ions for catalyzing the nucleotidyl transfer has been accepted for many decades [50, 62]. It has been proven for different DNA pol families as well as for different kingdoms of life [18, 19, 24, 45, 49]. Recent crystal structures of eukaryotic DNA pols from the B- (DNA pol δ [23]), X- (DNA pol β [63–65]) and Y-family (DNA pol ƞ [66–68]) revealed a third metal ion in the polymerase active site. For DNA pols ƞ and β this third metal ion is transiently coordinated by non-bridging oxygens of the α- and β-phosphates during and after product formation, respectively. In DNA pol ƞ, the third metal ion seems to facilitate product formation by lowering the energy barrier of the transition state [66–68] whereas in DNA pol β, this metal ion seems to facilitate the reverse reaction, pyrophosphorolysis. [63] In A- and B-family DNA pols, a conserved lysine residue is placed in the vicinity where the third metal ion binds in DNA pols ƞ and β.[69] This lysine is thought to be crucial for the protonation during the nucleotidyl transfer [69] but it may also serve in the same way as the third metal ion in the X- and Y-family. Thus, in DNA pol δ the third metal ion is found at a different position in the active site.[23] The ion is present prior to catalysis in the active complex and is coordinated by the γ-phosphate of the incoming triphosphate, the enzyme (E802, D608) and three water molecules.[23] Here, the third metal ion is believed to affect the incorporation of either right or wrong nucleotides, thereby modulating the catalytic efficiency. This third metal ion is also present in the complexes of KOD and 9°N DNA pols, which show a similar coordination as observed in DNA pol δ (S5 Fig). The metal coordinating amino acid side chains in KOD DNA pol (D404, E580) adopt a slightly different conformation compared to DNA pol δ (D608, E802) (S5 Fig), which may be due to the high B-factors observed for the metal ions bound in position A and C (43.3 and 40.7, respectively; metal ion B 20.2) in DNA pol δ, indicating high flexibility. The presence or absence of a third metal ion in some B-family DNA pols, may point to different modes of catalysis and/or discrimination. So far, the discrete role of the third metal ion in DNA pols δ, KOD, and 9°N is unclear, but may point to an evolutionary relationship between these B-family polymerases.[70]

A third metal ion, occupying a different place than the metal ion C in DNA pols KOD and δ, has also been found in one crystal structure of RB69 DNA pol, however, this third metal ion seems not to be involved in catalysis, due to the missing interaction with the dNTP.[71]

As we also obtained a 9°N DNA pol structure with only the two core metal ions (A and B), the coordination of the third metal ion might be weaker and is not essential for the closure of the finger domain and binding of the dNTP in the active site. Similar observations were made for metal ion A in A- and B-family DNA pols [24, 72] This may be due to water coordination of metal ions A and C compared to the exhaustively enzyme- and dNTP-coordinated metal ion B and in addition due to the missing 3’OH group in some structures. However, as shown for DNA pol δ, the nucleotidyl transfer can also proceed without a third metal ion.[23]

### Comparison to other DNA polymerases

The B-family DNA pols RB69, δ and KOD share high sequence similarity in the palm domain, the 5’-template interacting region, the sugar binding pocket and the metal coordinating residue region (D404 and D542) as well as in the minor groove sensing residue region. Additionally, DNA pols KOD and δ share conserved residues in the metal coordinating region of the third metal ion (E578) and an α-helix of the palm domain. The finger domain of the B-family polymerases exhibits conserved residues that interact with the triphosphate (K487, R460, K464) as well as the nascent base pair (S492, N491). The finger domain of KOD DNA pol is structurally closely related to the finger domain of DNA pol δ as both consist of two α-helices. The *O*-helix in KOD DNA pol (termed according to 9°N DNA pol [26]), which is the analogue to the *O*-helix in the A-family DNA pols, shows high sequence identity with the P-helix in DNA pol δ (S4 Fig). Furthermore, KOD and δ DNA pols show high sequence similarity in the exonuclease domain as well as in a patch in the N-terminal domain. Overall, the B-family DNA pols show high similarity in catalytically essential regions, but the high sequence conservation especially in the finger domain and the same metal coordination in the active site especially point to a close relationship between KOD DNA pol and DNA pol δ. Due to the simpler finger domain compared to RB69, KOD and DNA pol δ exhibit a broader gap between palm and finger domain, through which the pyrophosphate may diffuse [19, 23].

In case of DNA-protein interactions KOD DNA pol differs from other DNA pols, as it exhibits more interactions with the 3’-template region compared to DNA pols δ, RB69 and KTQ. These direct strong hydrogen bonds may contribute to a good stabilization of the template strand and the bound DNA duplex and may thereby account for the high processivity of KOD DNA pol.[57] Additionally, all B-family DNA pols exhibit a crevice with an electropositive potential between the β-hairpin and the N-terminal domain, which may interact with the single stranded template, and thereby further stabilize the enzyme binding to the DNA strand. This might further contribute to the protein’s proficiencies in processivity.

### B-family polymerases and their application in biotechnology

As the incorporation of modified triphosphates is crucial for various biochemical approaches like next generation sequencing [2] the ability of thermostable A- and B-family DNA pols to incorporate modified nucleotides was extensively studied. It was shown that B-family DNA pols accept nucleotides with the modification pointing into the major groove (C5 for pyrimidines and 7-deaza for purines) often better than the A-family DNA pols.[12, 16, 17, 51–56] The B-family DNA pols exhibit broader substrate tolerance [16, 17] and incorporate multiple modified nucleotides [51, 52, 54] into the nascent DNA strand. So far, all structural knowledge concerning the incorporation of C5 modified pyrimidine and 7-deaza modified purine dNTPs is derived from the A-family DNA pol KlenTaq.[42, 58, 73–75] These structures revealed certain aspects: DNA adopting A-form close to the active site, the small channel between the thumb and finger domain as well as amino acid side chains that can form interactions with the major groove of the DNA.[49, 58] The interaction via R660 with the nascent major groove seems to be specific for dGTP in KlenTaq DNA pol. [49] In comparison, the DNA in KOD DNA pol adopts B-form, which leads to a wider major groove compared to A-form DNA.[76] KOD DNA pol also has two large channels between its thumb and palm domain, where modifications attached at the nucleobase could be accommodated (S10 Fig). Additionally, in the KOD DNA pol structure no major groove interacting amino acid side chains are visible. KOD DNA pol does not exhibit an arginine residue that is analogous to R660 in KlenTaq DNA pol. Furthermore, the arginine residues in KOD DNA pol, which interact with the primer strand close to the active site (R606, R613) do not position into the major groove, as this would most likely need significant conformational changes. Only R668 of the thumb domain may interact with modifications positioned in the major groove.

Overall, the missing major groove interacting amino acids, the wider major groove in B-form DNA, the larger channels in KOD DNA pol as well as the shape of the pocket formed around the bound dATP with its good solvent accessibility of the N7 atom (Fig 3B), provide more space for accommodating nucleotide modifications than in the A-family DNA pols. This might be causative for the superiority of archaeal B-family DNA pols in processing modified nucleotides.

The proficient acceptance of modified nucleotides by DNA pols from archaea resulted in the use of a 9°N DNA pol mutant A485L in next generation sequencing.[1] The A485 is embedded in the O-helix of the finger domain, pointing towards the I-helix residues M329, Glu330 and l333 of the N-terminal domain. Thereby, the A485L mutation may cause hydrophobic interactions with the neighboring α-helix that could affect the closure of the finger domain.

### Accession numbers

#### RCSB Protein Data Bank

Coordinate and structure factors for the KOD DNA pol have been deposited with the accession code 5OMF, for the 9°N DNA pol with three metal ions in the active site with the accession code 5OMQ and for 9°N with two metal ions in the active site with the accession code 5OMV.

#### SBGrid Data Bank

Raw crystallographic data were deposited with the SBGrid data bank as doi:10.15785/SBGRID/462 for the KOD DNA pol, as doi:10.15785/SBGRID/479 for the 9°N DNA pol with three metal ions in the active site and as doi:10.15785/SBGRID/480 for the 9°N DNA pol with two metal ions in the active site.

## Supporting information

S1 Table Data processing and refinement statistics. (PDF)

S1 Fig Activity assay of 9°N and KOD DNA pol. The activity of the enzymes was determined by amplifying a 1044 bp long part of the KOD DNA pol gene using 1x Phusion HF Buffer (New England Biolabs), 0.2 ng/μL template (pET24a vector with the KOD DNA pol gene), 500 nM forward primer (5’-d(TTTGCACTGGGTCGTGATG)), 500 nM reverse primer (5’-d(CAGTTCCAGTGCACCCGGC)), 200 μM dNTPs and DNA pol (5 nM KOD DNA pol, 5 nM 9°N DNA pol or 2 units Phusion HF (New England Biolabs, 2000 units/mL)), 28 μL H_2_O; lane 1: 9°N DNA pol, lane 2: KOD DNA pol, lane 3: Phusion DNA pol, lane 4: without polymerase, lane 5: 1 kbp DNA ladder.
(PDF)

S2 Fig Movement of amino acids interacting with the incoming dATP between the open and closed conformation. The finger domain of the binary (black) and ternary (white) complex were superimposed, showing the movement of the amino acids interacting with the triphosphate measured from the Cα-atom (shown as sphere) of the respective amino acid and given in Å, Q483 (green), R460 (yellow), Q461 (blue), K464 (cyan), K487 (pink) and N491 (orange), the dATP of the ternary complex is shown as grey stick with grey surface. Due to the rotation of the finger domain the amino acids located closer to the tip of the finger domain undergo a bigger movement upon binding of the dATP, see D472 (white) with a movement of the Cα-atom of 14.6 Å.
(PDF)

S3 Fig Position of the β-hairpin in DNA pols KOD, RB69 and δ. After superimposition of the polymerases, the β-hairpins of DNA pols KOD, RB69 and δ are shown in grey, purple and red, respectively. The DNA is shown in grey, purple and red, respectively. For orientation, the domains of DNA pol KOD are shown color-coded in transparent.
(PDF)

S4 Fig Sequence comparison of DNA pols KOD, δ and RB69. KOD DNA pol is shown as a grey surface with the bound DNA in pink. The amino acids which are identical between DNA pols KOD (1^st^ row) and δ (2^nd^ row) are highlighted in blue. Sequence patches of high identity according to sequence alignments using PROMALSS3D between DNA pols KOD, δ and RB69 (3^rd^ row) are shown for the different domains alongside the figures with blue letters indicating identical amino acids and orange letters indicating homologue amino acids. The residues needed for metal coordination in the exonuclease and polymerase active site are highlighted by grey boxes.
(PDF)

S5 Fig Active site of DNA pol δ. (A) The three Ca^2+^ ions (green) in the active site of DNA pol δ are coordinated by residues of the palm domain (cyan). Metal ion A is coordinated by the α-phosphate of the dATP (pink), two water molecules and D764. Metal ion B is coordinated by the α-, β- and γ-phosphate, D764, D608 and F609. Metal ion C is coordinated by the γ-phosphate, D608, E802 and three water molecules. One water molecule is coordinated by E800. The dCTP makes direct interactions with the conserved finger domain residues (yellow) N705, K701 and R674 as well as water mediated interactions with K678. (B) Superimposition of the metal coordination in KOD DNA pol (dATP, waters and metal ions are shown in transparent) and pol δ. The coordinating amino acid side chains are colored in blue for KOD DNA pol and cyan for pol δ, showing the slightly different conformations of D404 and E580 for KOD DNA pol compared to D608 and E802 for DNA pol δ.
(PDF)

S6 Fig Polymerase active site of 9°N DNA pol with bound dATP. (A) The metal ions are coordinated by residues of the palm domain (cyan). Metal ion A (Mg^2+^, green) is coordinated by two water molecules, D542, the α-phosphate of the dATP (pink) and D404. Metal ion B (Mn^2+^, purple) is coordinated by the α-, β- and γ-phosphate, D404, F405 and D542. Metal ion C (Mg^2+^, green) is coordinated by the γ-phosphate, E580, F405, D404 and three water molecules, whereof one molecule is coordinated by E578 and the other by K464 of the finger domain (yellow). The dATP makes further direct contacts with conserved residues of the finger domain (yellow), N491, K487 and R460 as well as a water mediated contact to K464. (B) Superimposition of the metal coordination in KOD DNA pol (shown in transparent grey) and 9°N DNA pol. The coordinating amino acid side chains show the exact same coordination in both polymerases. (C) Superimposition of the two 9°N structures with three metal ions (dATP, waters and metal ions are shown in transparent, amino acids are shown in cyan) and with two metal ions (amino acids are shown in grey). The side chains of E580 and E578 that coordinate the third metal ion adopt different conformations in absence of the third ion.
(PDF)

S7 Fig Interaction patterns of DNA pol δ (PDB ID: 3IAY), RB69 DNA pol (PDB ID: 3NCI) and KlenTaq DNA pol (PDB ID: 3RTV). The interactions between the enzyme and the respective dNTP as well as between the enzyme and the template/ primer strand were assigned according to their strengths (see legend). Stacking interactions with the dNTP are shown as black dashes.
(PDF)

S8 Fig Electropotential map of DNA pols δ and RB69. The electropotential is shown from +6 (red) to -6 (blue) k_B_T/e (T = 310 K). The primer is shown in pink, the template in violet and the dNTP as yellow sticks. DNA pols δ and RB69 exhibit a positively charged crevice reaching from the thumb domain along the palm and β-hairpin upwards to the N-terminal domain. In the crevice between the N-terminal and exonuclease domain, the single stranded template may bind. Additionally, DNA pol δ shows two positively charged patches at the exonuclease and thumb domain.
(PDF)

S9 Fig Channel volumes of DNA pols δ and RB69. The channel volumes were calculated with 3V algorithm for DNA pols δ (A and B) and RB69 (C and D). The protein is shown as grey surface, the primer in cyan and the template in blue. The bound dNTP is shown in magenta. A and C show the location within the enzyme, B and D the channels in respect to the DNA and triphosphate. For RB69 DNA pol the channel between the β-hairpin and the N-terminal domain shows an additional “outer” channel, which is not directly located at the DNA and can thereby not be used for the positioning of modifications. The “outer” channel occupies the electronegative crevice in which the single stranded template is may bind.
(PDF)

S10 Fig Modelling of a modified nucleotide into KOD DNA pol. The 7-(N-(10-hydroxydecanoyl)-aminopentinyl)-7-deaza-2-dATP (dATP*) (PDB Code: 0L3) was modelled into the active site of KOD DNA pol. The nucleotide moiety of the dATP* was superimposed with the dATP of the KOD DNA pol structure and the linker was modelled in two conformations (green and pink sticks) into the free space within the enzyme using COOT [2]. (A) View onto the Hoogsteen side of the dATP* showing the linker modelled in green pointing towards the finger and palm domain. (B) Rotation of approx. 130° showing the linker modelled in pink pointing towards the thumb domain and the β-hairpin.
(PDF)
