# Supplementary material for: Crystal structures of ternary complexes of archaeal B-family DNA polymerases
Source: PLoS One. 2017 Dec 6;12(12):e0188005. doi: 10.1371/journal.pone.0188005 (PMC5718519; doi:10.1371/journal.pone.0188005)
Supplement: S1 Table — (PDF) [file pone.0188005.s001.pdf]

Crystallographic data

S1 Table. Data processing and refinement statistics.

**Data set name**	KOD DNA pol	9°N DNA pol with 2metal ions	9°N DNA pol with 3 metal ions
**PDB ID**	5OMF	5OMV	5OMQ
Date of data collection	10.08.2016	07.12.2016	07.12.2016
Wavelength (Å)	1.0	1.0	1.0
Detector	EIGER16M	PILATUS	PILATUS
Space group	$P2_12_12$	$P2_12_12_1$	$P2_12_12_1$
**Cell dimensions**			
a, b, c (Å)	107.95, 147.57, 71.18	66.7, 95.0, 158.7	66.8, 93.7, 158.2
α, β, γ (°)	90.00, 90.00, 90.00	90, 90, 90	90, 90, 90
Resolution (Å)	49.19-2.09 (2.22-2.09)	47.5-2.0 (2.12-2.00)	46.8-2.2 (2.33-2.20)
No. of total reflections	455650 (68286)	459321 (74747)	337245 (53785)
No. of unique reflections	127923 (20049)	130653 (21057)	97025 (15527)
R_{meas} (%)	18.5 (172.0)	14.2 (360.3)	10.8 (154.9)
I / σ	6.37 (0.81)	7.88 (0.37)	8.62 (0.92)
Completeness (%)	98.8 (95.8)	99.7 (99.6)	99.6 (98.8)
Redundancy	3.56 (3.41)	3.5 (3.5)	3.5 (3.5)
$CC_{1/2}$ (%)	99.3 (28.5)	99.7 (11.1)	99.8 (47.6)
Isa [1]	32.30	36.0	22.8
**Refinement**			
Resolution (Å)	46.266-2.092	47.482-2.003	46.839-2.199
No. of reflections	127892	130623	96987
R_{work}/ R_{free}	19.53/23.35	18.94/23.32	19.30/24.83
Coordinate error	0.32	0.39	0.36
No. of atoms			
Protein	12455	6208	6208
DNA (p/t/dATP)	371/447/46	239/309/42	239/288/42
Water	269	179	90
Average B-factors (Å)			
Protein	58.61	57.71	62.09
DNA (p/t/dATP)	62.61/57.05/34.30	48.77/56.39/41.42	55.39/56.85/43.03
Water	46.24	53.77	51.10
R.m.s deviations			
Bond lengths (Å)	0.002	0.008	0.008
Bond angles (°)	0.527	0.924	0.934
Ramachandran plot (%)			
Favored	96.55	97.22	97.35
Allowed	3.18	2.78	2.65
Outlier	0.27	0.00	0.00

Values in parentheses are for highest-resolution shell. p: primer, t: template

1. Diederichs K. Quantifying instrument errors in macromolecular X-ray data sets. Acta Crystallogr, Sect D: Biol Crystallogr. 2010;66(6):733-40. doi: doi:10.1107/S0907444910014836.
