# Supplementary material for: Crystal structures of ternary complexes of archaeal B-family DNA polymerases
Source: PLoS One. 2017 Dec 6;12(12):e0188005. doi: 10.1371/journal.pone.0188005 (PMC5718519; doi:10.1371/journal.pone.0188005)
Supplement: S1 Fig — The activity of the enzymes was determined by amplifying a 1044 bp long part of the KOD DNA pol gene using 1x Phusion HF Buffer (New England Biolabs), 0.2 ng/μL template (pET24a vector with the KOD DNA pol gene), 500 nM forward primer (5’-d(TTTGCACTGGGTCGTGATG)), 500 nM reverse primer (5’-d(CAGTTCCAGTGCACCCGGC)), 200 μM dNTPs and DNA pol (5 nM KOD DNA pol, 5 nM 9°N DNA pol or 2 units Phusion HF (New England Biolabs, 2000 units/mL)), 28 μL H2O; lane 1: 9°N DNA pol, lane 2: KOD DNA pol, lane 3: Phusion DNA pol, lane 4: without polymerase, lane 5: 1 kbp DNA ladder. (PDF) [file pone.0188005.s002.pdf]

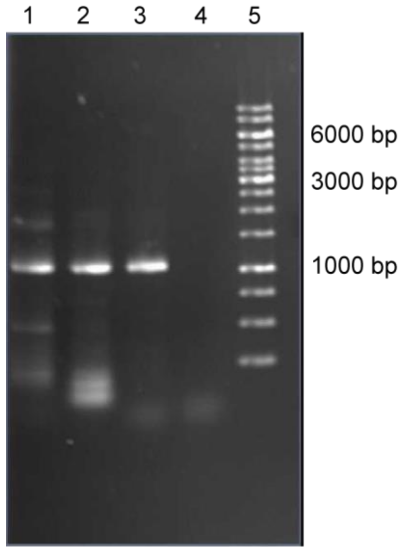


**S1 Fig. Activity assay of 9°N and KOD DNA pol.** The activity of the enzymes was determined by amplifying a 1044 bp long part of the KOD DNA pol gene using 1x Phusion HF Buffer (New England Biolabs), 0.2 ng/µL template (pET24a vector with the KOD DNA pol gene), 500 nM forward primer (5'-d(TTTGCACTGGGTCGTGATG)), 500 nM reverse primer (5'-d(CAGTTCCAGTGCACCCGGC)), 200 µM dNTPs and DNA pol (5 nM KOD DNA pol, 5 nM 9°N DNA pol or 2 units Phusion HF (New England Biolabs, 2000 units/mL)), 28 µL $H_2O$; lane 1: 9°N DNA pol, lane 2: KOD DNA pol, lane 3: Phusion DNA pol, lane 4: without polymerase, lane 5: 1 kbp DNA ladder.
