# Supplementary material for: Crystal structures of ternary complexes of archaeal B-family DNA polymerases
Source: PLoS One. 2017 Dec 6;12(12):e0188005. doi: 10.1371/journal.pone.0188005 (PMC5718519; doi:10.1371/journal.pone.0188005)
Supplement: S2 Fig — The finger domain of the binary (black) and ternary (white) complex were superimposed, showing the movement of the amino acids interacting with the triphosphate measured from the Cα-atom (shown as sphere) of the respective amino acid and given in Å, Q483 (green), R460 (yellow), Q461 (blue), K464 (cyan), K487 (pink) and N491 (orange), the dATP of the ternary complex is shown as grey stick with grey surface. Due to the rotation of the finger domain the amino acids located closer to the tip of the finger domain undergo a bigger movement upon binding of the dATP, see D472 (white) with a movement of the Cα-atom of 14.6 Å. (PDF) [file pone.0188005.s003.pdf]

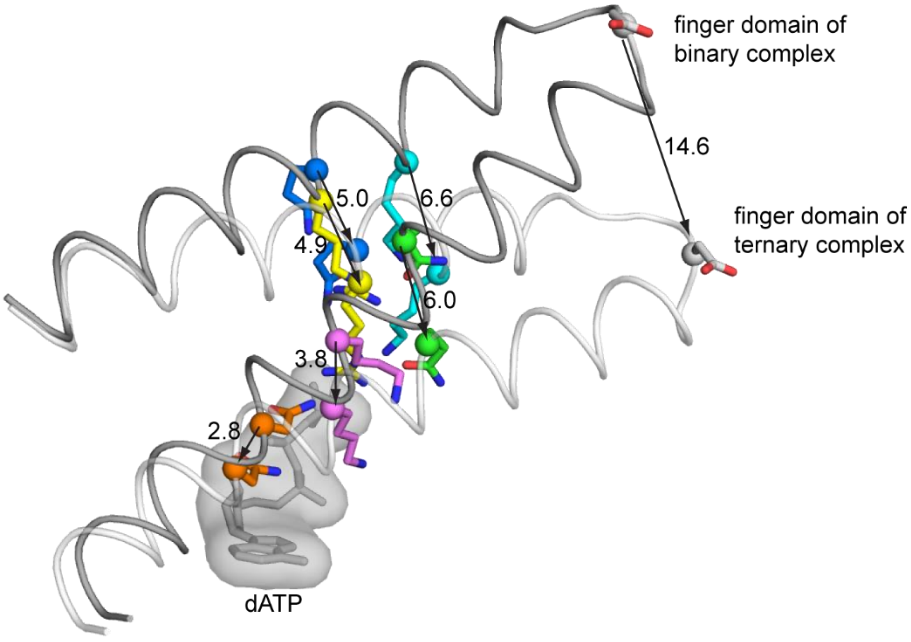


**S2 Fig. Movement of amino acids interacting with the incoming dATP between the open and closed conformation.** The finger domain of the binary (black) and ternary (white) complex were superimposed, showing the movement of the amino acids interacting with the triphosphate measured from the Cα-atom (shown as sphere) of the respective amino acid and given in Å, Q483 (green), R460 (yellow), Q461 (blue), K464 (cyan), K487 (pink) and N491 (orange), the dATP of the ternary complex is shown as grey stick with grey surface. Due to the rotation of the finger domain the amino acids located closer to the tip of the finger domain undergo a bigger movement upon binding of the dATP, see D472 (white) with a movement of the Cα-atom of 14.6 Å.
