# Supplementary material for: Crystal structures of ternary complexes of archaeal B-family DNA polymerases
Source: PLoS One. 2017 Dec 6;12(12):e0188005. doi: 10.1371/journal.pone.0188005 (PMC5718519; doi:10.1371/journal.pone.0188005)
Supplement: S3 Fig — After superimposition of the polymerases, the β-hairpins of DNA pols KOD, RB69 and δ are shown in grey, purple and red, respectively. The DNA is shown in grey, purple and red, respectively. For orientation, the domains of DNA pol KOD are shown color-coded in transparent. (PDF) [file pone.0188005.s004.pdf]

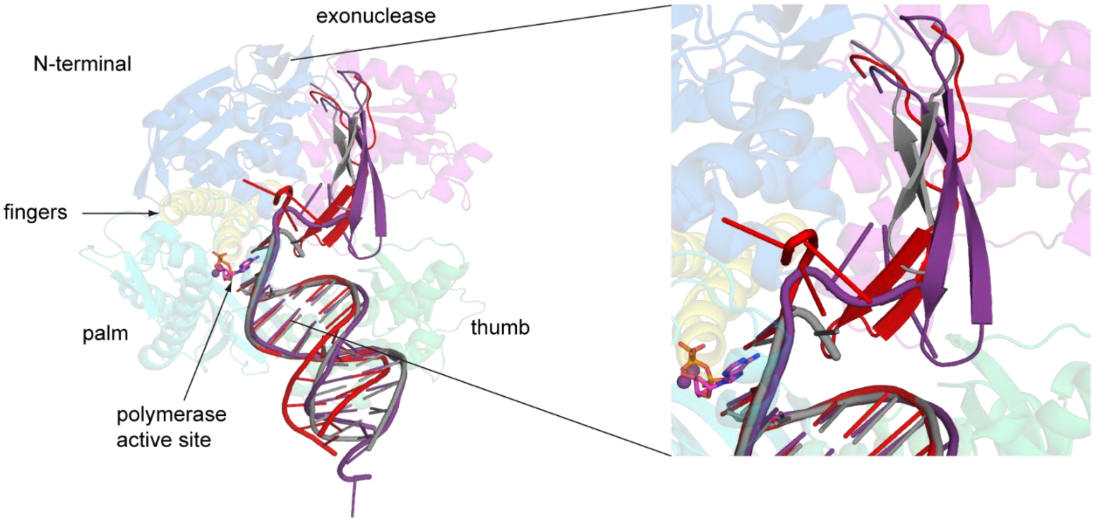

S3 Fig. Position of the β-hairpin in DNA pols KOD, RB69 and δ. After superimposition of the polymerases, the β-hairpins of DNA pols KOD, RB69 and δ are shown in grey, purple and red, respectively. The DNA is shown in grey, purple and red, respectively. For orientation, the domains of DNA pol KOD are shown color-coded in transparent.
