# Supplementary material for: Crystal structures of ternary complexes of archaeal B-family DNA polymerases
Source: PLoS One. 2017 Dec 6;12(12):e0188005. doi: 10.1371/journal.pone.0188005 (PMC5718519; doi:10.1371/journal.pone.0188005)
Supplement: S4 Fig — KOD DNA pol is shown as a grey surface with the bound DNA in pink. The amino acids which are identical between DNA pols KOD (1st row) and δ (2nd row) are highlighted in blue. Sequence patches of high identity according to sequence alignments using PROMALSS3D between DNA pols KOD, δ and RB69 (3rd row) are shown for the different domains alongside the figures with blue letters indicating identical amino acids and orange letters indicating homologue amino acids. The residues needed for metal coordination in the exonuclease and polymerase active site are highlighted by grey boxes. (PDF) [file pone.0188005.s005.pdf]

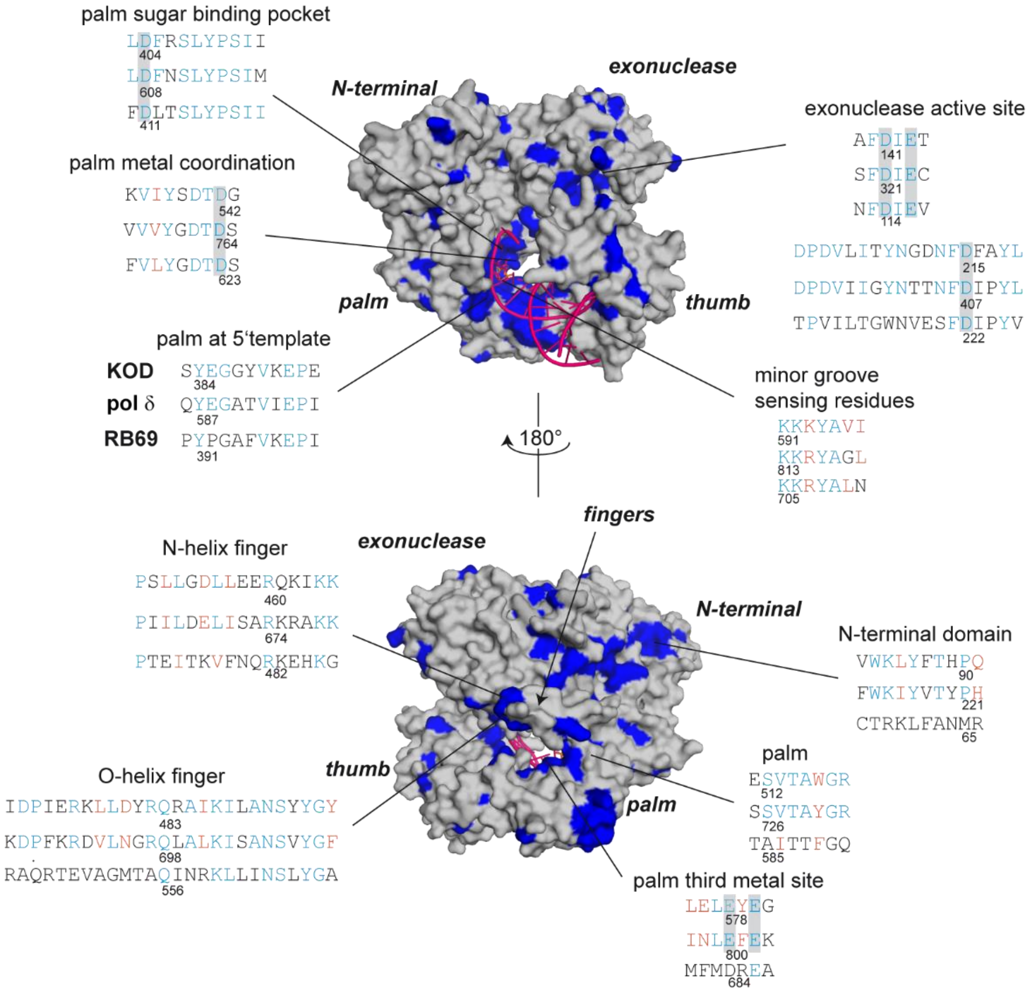

S4 Fig. Sequence comparison of DNA pols KOD, δ and RB69. KOD DNA pol is shown as a grey surface with the bound DNA in pink. The amino acids which are identical between DNA pols KOD (1st row) and δ (2nd row) are highlighted in blue. Sequence patches of high identity according to sequence alignments using PROMALSS3D between DNA pols KOD, δ and RB69 (3rd row) are shown for the different domains alongside the figures with blue letters indicating identical amino acids and orange letters indicating homologue amino acids. The residues needed for metal coordination in the exonuclease and polymerase active site are highlighted by grey boxes.
