# Supplementary material for: Crystal structures of ternary complexes of archaeal B-family DNA polymerases
Source: PLoS One. 2017 Dec 6;12(12):e0188005. doi: 10.1371/journal.pone.0188005 (PMC5718519; doi:10.1371/journal.pone.0188005)
Supplement: S5 Fig — (A) The three Ca2+ ions (green) in the active site of DNA pol δ are coordinated by residues of the palm domain (cyan). Metal ion A is coordinated by the α-phosphate of the dATP (pink), two water molecules and D764. Metal ion B is coordinated by the α-, β- and γ-phosphate, D764, D608 and F609. Metal ion C is coordinated by the γ-phosphate, D608, E802 and three water molecules. One water molecule is coordinated by E800. The dCTP makes direct interactions with the conserved finger domain residues (yellow) N705, K701 and R674 as well as water mediated interactions with K678. (B) Superimposition of the metal coordination in KOD DNA pol (dATP, waters and metal ions are shown in transparent) and pol δ. The coordinating amino acid side chains are colored in blue for KOD DNA pol and cyan for pol δ, showing the slightly different conformations of D404 and E580 for KOD DNA pol compared to D608 and E802 for DNA pol δ. (PDF) [file pone.0188005.s006.pdf]

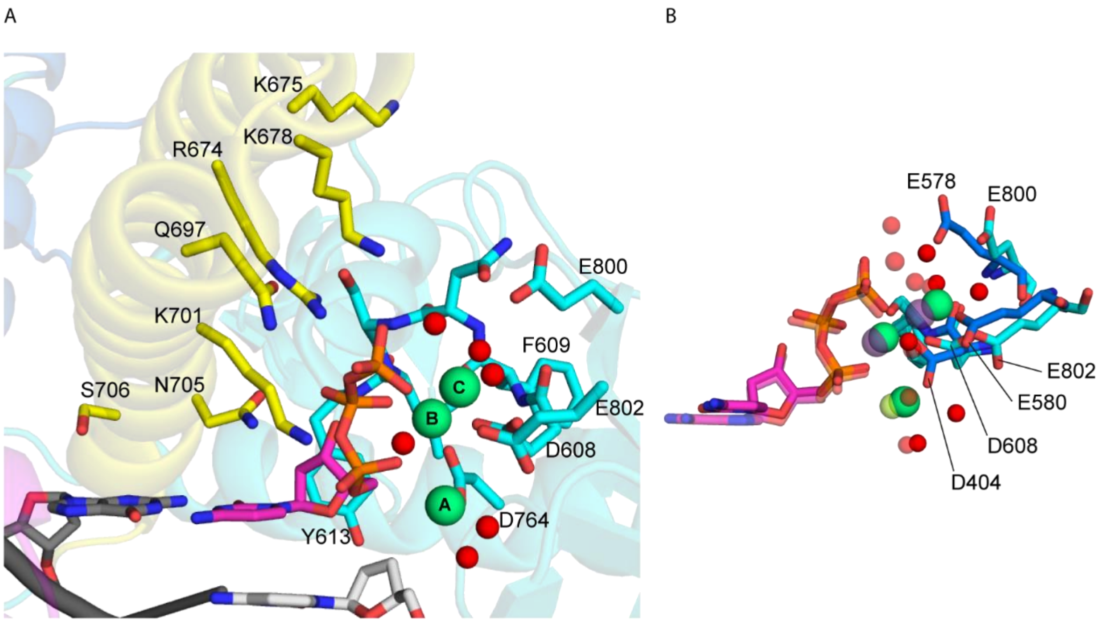

S5 Fig. Active site of DNA pol δ. (A) The three Ca^{2+} ions (green) in the active site of DNA pol δ are coordinated by residues of the palm domain (cyan). Metal ion A is coordinated by the α-phosphate of the dATP (pink), two water molecules and D764. Metal ion B is coordinated by the α-, β- and γ-phosphate, D764, D608 and F609. Metal ion C is coordinated by the γ-phosphate, D608, E802 and three water molecules. One water molecule is coordinated by E800. The dCTP makes direct interactions with the conserved finger domain residues (yellow) N705, K701 and R674 as well as water mediated interactions with K678. (B) Superimposition of the metal coordination in KOD DNA pol (dATP, waters and metal ions are shown in transparent) and pol δ. The coordinating amino acid side chains are colored in blue for KOD DNA pol and cyan for pol δ, showing the slightly different conformations of D404 and E580 for KOD DNA pol compared to D608 and E802 for DNA pol δ.
