# Supplementary material for: Crystal structures of ternary complexes of archaeal B-family DNA polymerases
Source: PLoS One. 2017 Dec 6;12(12):e0188005. doi: 10.1371/journal.pone.0188005 (PMC5718519; doi:10.1371/journal.pone.0188005)
Supplement: S6 Fig — (A) The metal ions are coordinated by residues of the palm domain (cyan). Metal ion A (Mg2+, green) is coordinated by two water molecules, D542, the α-phosphate of the dATP (pink) and D404. Metal ion B (Mn2+, purple) is coordinated by the α-, β- and γ-phosphate, D404, F405 and D542. Metal ion C (Mg2+, green) is coordinated by the γ-phosphate, E580, F405, D404 and three water molecules, whereof one molecule is coordinated by E578 and the other by K464 of the finger domain (yellow). The dATP makes further direct contacts with conserved residues of the finger domain (yellow), N491, K487 and R460 as well as a water mediated contact to K464. (B) Superimposition of the metal coordination in KOD DNA pol (shown in transparent grey) and 9°N DNA pol. The coordinating amino acid side chains show the exact same coordination in both polymerases. (C) Superimposition of the two 9°N structures with three metal ions (dATP, waters and metal ions are shown in transparent, amino acids are shown in cyan) and with two metal ions (amino acids are shown in grey). The side chains of E580 and E578 that coordinate the third metal ion adopt different conformations in absence of the third ion. (PDF) [file pone.0188005.s007.pdf]

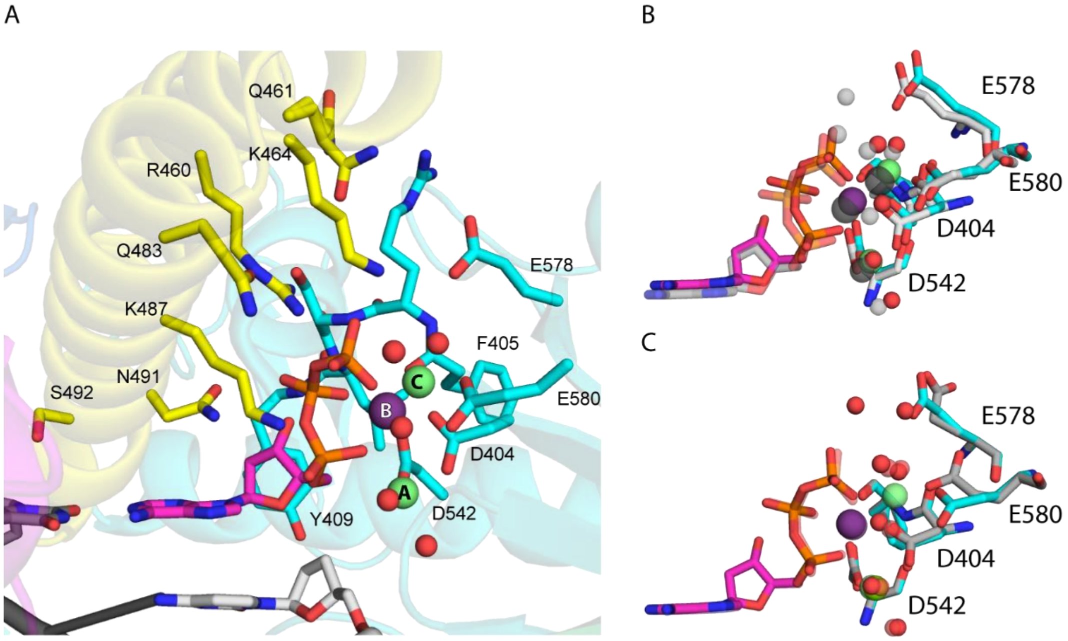

S6 Fig. Polymerase active site of 9°N DNA pol with bound dATP. (A) The metal ions are coordinated by residues of the palm domain (cyan). Metal ion A (Mg^{2+}, green) is coordinated by two water molecules, D542, the α-phosphate of the dATP (pink) and D404. Metal ion B (Mn^{2+}, purple) is coordinated by the α-, β- and γ-phosphate, D404, F405 and D542. Metal ion C (Mg^{2+}, green) is coordinated by the γ-phosphate, E580, F405, D404 and three water molecules, whereof one molecule is coordinated by E578 and the other by K464 of the finger domain (yellow). The dATP makes further direct contacts with conserved residues of the finger domain (yellow), N491, K487 and R460 as well as a water mediated contact to K464. (B) Superimposition of the metal coordination in KOD DNA pol (shown in transparent grey) and 9°N DNA pol. The coordinating amino acid side chains show the exact same coordination in both polymerases. (C) Superimposition of the two 9°N structures with three metal ions (dATP, waters and metal ions are shown in transparent, amino acids are shown in cyan) and with two metal ions (amino acids are shown in grey). The side chains of E580 and E578 that coordinate the third metal ion adopt different conformations in absence of the third ion.
