# Supplementary material for: Crystal structures of ternary complexes of archaeal B-family DNA polymerases
Source: PLoS One. 2017 Dec 6;12(12):e0188005. doi: 10.1371/journal.pone.0188005 (PMC5718519; doi:10.1371/journal.pone.0188005)
Supplement: S7 Fig — The interactions between the enzyme and the respective dNTP as well as between the enzyme and the template/ primer strand were assigned according to their strengths (see legend). Stacking interactions with the dNTP are shown as black dashes. (PDF) [file pone.0188005.s008.pdf]

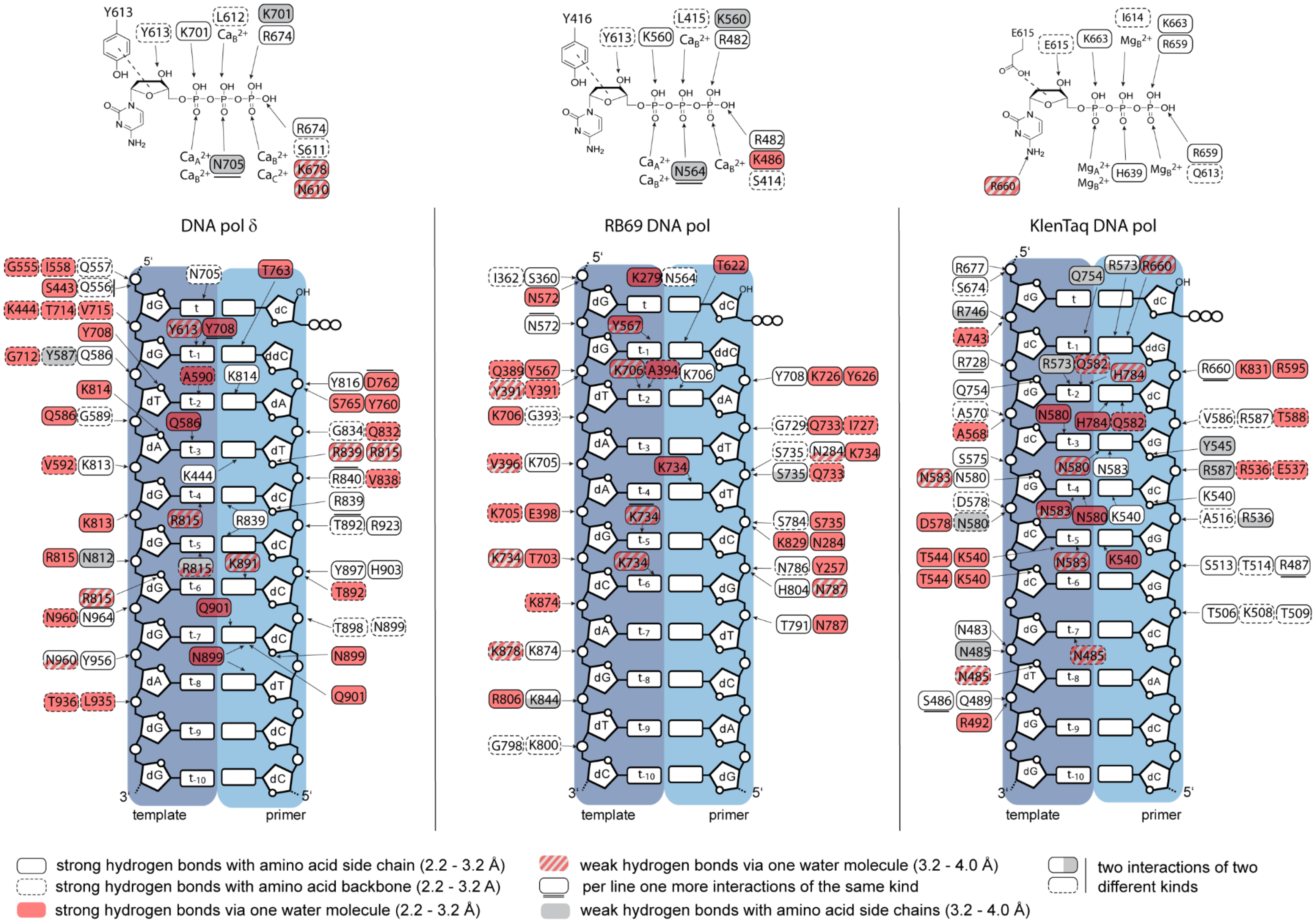
DNA pol δ
RB69 DNA pol
KlenTaq DNA pol
template
primer
strong hydrogen bonds with amino acid side chain (2.2 - 3.2 Å)
strong hydrogen bonds with amino acid backbone (2.2 - 3.2 A)
strong hydrogen bonds via one water molecule (2.2 - 3.2 Å)
weak hydrogen bonds via one water molecule (3.2 - 4.0 Å)
per line one more interactions of the same kind
weak hydrogen bonds with amino acid side chains (3.2 - 4.0 Å)
two interactions of two different kinds

S7 Fig. Interaction patterns of DNA pol δ (PDB ID: 3IAY), RB69 DNA pol (PDB ID: 3NCI) and KlenTaq DNA pol (PDB ID: 3RTV). The interactions between the enzyme and the respective dNTP as well as between the enzyme and the template/ primer strand were assigned according to their strengths (see legend). Stacking interactions with the dNTP are shown as black dashes.
