# Supplementary material for: Crystal structures of ternary complexes of archaeal B-family DNA polymerases
Source: PLoS One. 2017 Dec 6;12(12):e0188005. doi: 10.1371/journal.pone.0188005 (PMC5718519; doi:10.1371/journal.pone.0188005)
Supplement: S8 Fig — The electropotential is shown from +6 (red) to -6 (blue) kBT/e (T = 310 K). The primer is shown in pink, the template in violet and the dNTP as yellow sticks. DNA pols δ and RB69 exhibit a positively charged crevice reaching from the thumb domain along the palm and β-hairpin upwards to the N-terminal domain. In the crevice between the N-terminal and exonuclease domain, the single stranded template may bind. Additionally, DNA pol δ shows two positively charged patches at the exonuclease and thumb domain. (PDF) [file pone.0188005.s009.pdf]

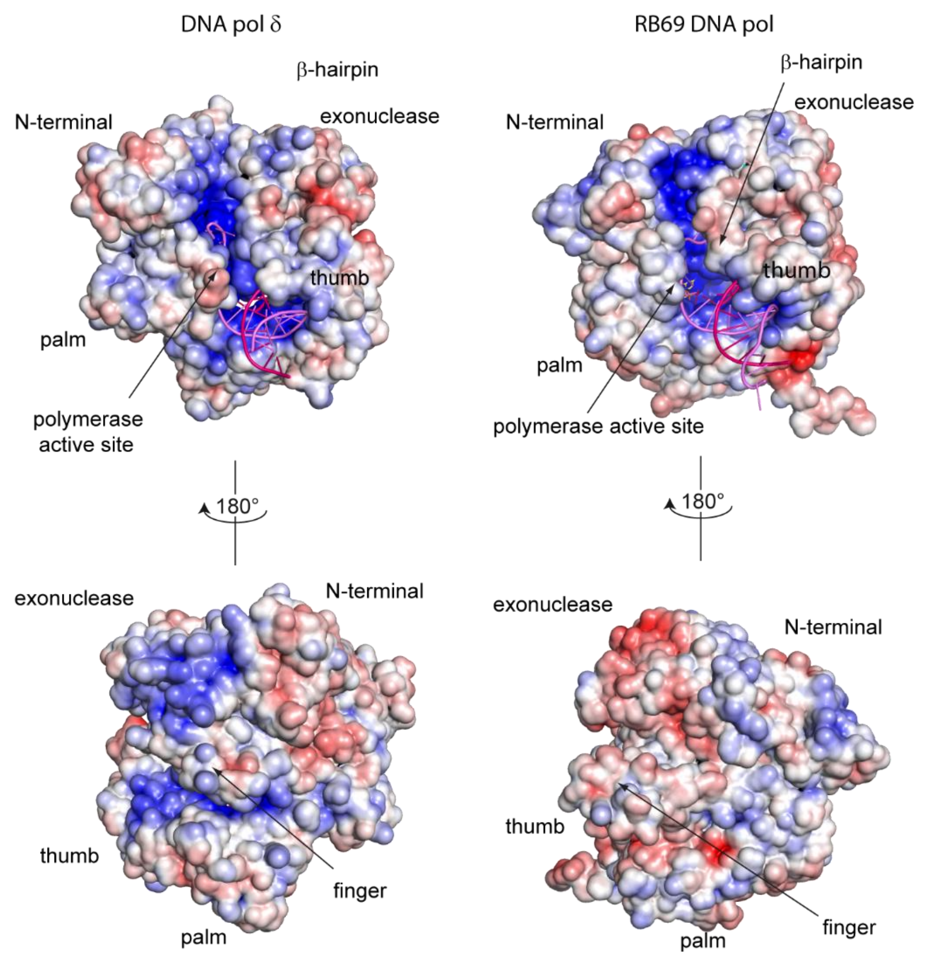

S8 Fig. Electropotential map of DNA pols δ and RB69. The electropotential is shown from +6 (red) to -6 (blue) k_BT/e (T=310 K). The primer is shown in pink, the template in violet and the dNTP as yellow sticks. DNA pols δ and RB69 exhibit a positively charged crevice reaching from the thumb domain along the palm and β-hairpin upwards to the N-terminal domain. In the crevice between the N-terminal and exonuclease domain, the single stranded template may bind. Additionally, DNA pol δ shows two positively charged patches at the exonuclease and thumb domain.
