# Supplementary material for: Crystal structures of ternary complexes of archaeal B-family DNA polymerases
Source: PLoS One. 2017 Dec 6;12(12):e0188005. doi: 10.1371/journal.pone.0188005 (PMC5718519; doi:10.1371/journal.pone.0188005)
Supplement: S9 Fig — The channel volumes were calculated with 3V algorithm for DNA pols δ (A and B) and RB69 (C and D). The protein is shown as grey surface, the primer in cyan and the template in blue. The bound dNTP is shown in magenta. A and C show the location within the enzyme, B and D the channels in respect to the DNA and triphosphate. For RB69 DNA pol the channel between the β-hairpin and the N-terminal domain shows an additional “outer” channel, which is not directly located at the DNA and can thereby not be used for the positioning of modifications. The “outer” channel occupies the electronegative crevice in which the single stranded template is may bind. (PDF) [file pone.0188005.s010.pdf]

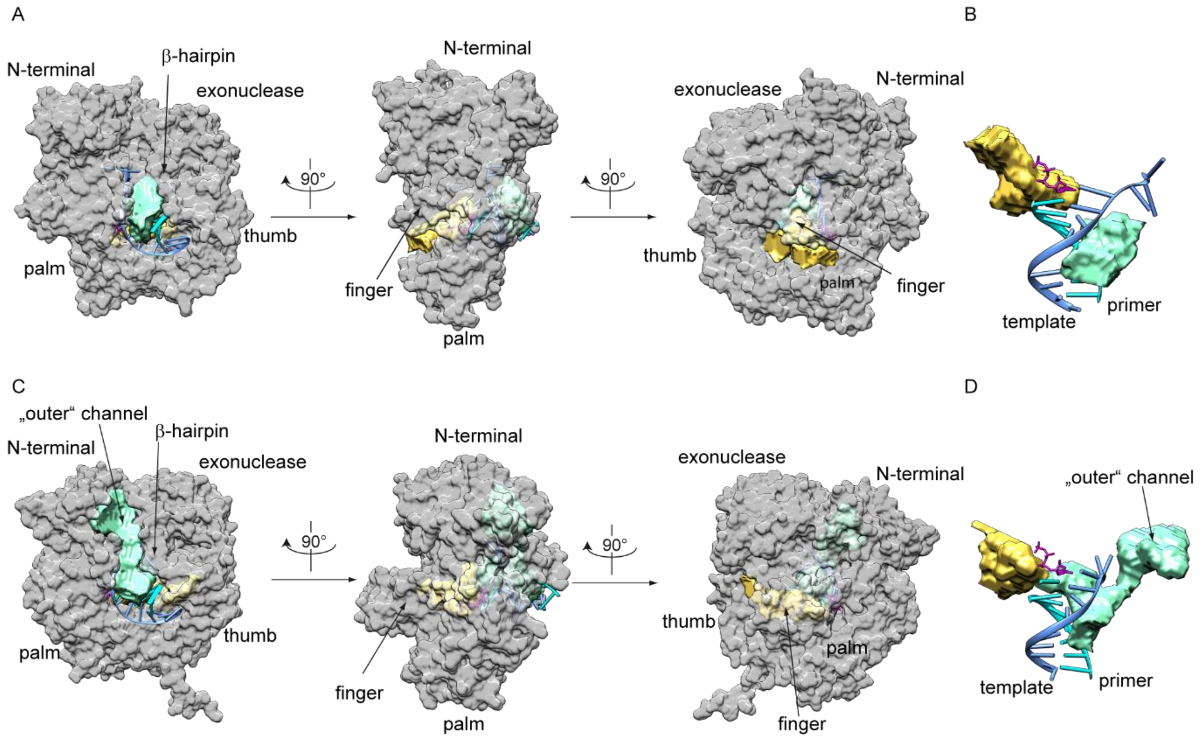

S9 Fig. Channel volumes of DNA pols δ and RB69. The channel volumes were calculated with 3V algorithm for DNA pols δ (A and B) and RB69 (C and D). The protein is shown as grey surface, the primer in cyan and the template in blue. The bound dNTP is shown in magenta. A and C show the location within the enzyme, B and D the channels in respect to the DNA and triphosphate. For RB69 DNA pol the channel between the β-hairpin and the N-terminal domain shows an additional “outer” channel, which is not directly located at the DNA and can thereby not be used for the positioning of modifications. The “outer” channel occupies the electronegative crevice in which the single stranded template is may bind.
