# Supplementary material for: Crystal structures of ternary complexes of archaeal B-family DNA polymerases
Source: PLoS One. 2017 Dec 6;12(12):e0188005. doi: 10.1371/journal.pone.0188005 (PMC5718519; doi:10.1371/journal.pone.0188005)
Supplement: S10 Fig — The 7-(N-(10-hydroxydecanoyl)-aminopentinyl)-7-deaza-2-dATP (dATP*) (PDB Code: 0L3) was modelled into the active site of KOD DNA pol. The nucleotide moiety of the dATP* was superimposed with the dATP of the KOD DNA pol structure and the linker was modelled in two conformations (green and pink sticks) into the free space within the enzyme using COOT [2]. (A) View onto the Hoogsteen side of the dATP* showing the linker modelled in green pointing towards the finger and palm domain. (B) Rotation of approx. 130° showing the linker modelled in pink pointing towards the thumb domain and the β-hairpin. (PDF) [file pone.0188005.s011.pdf]

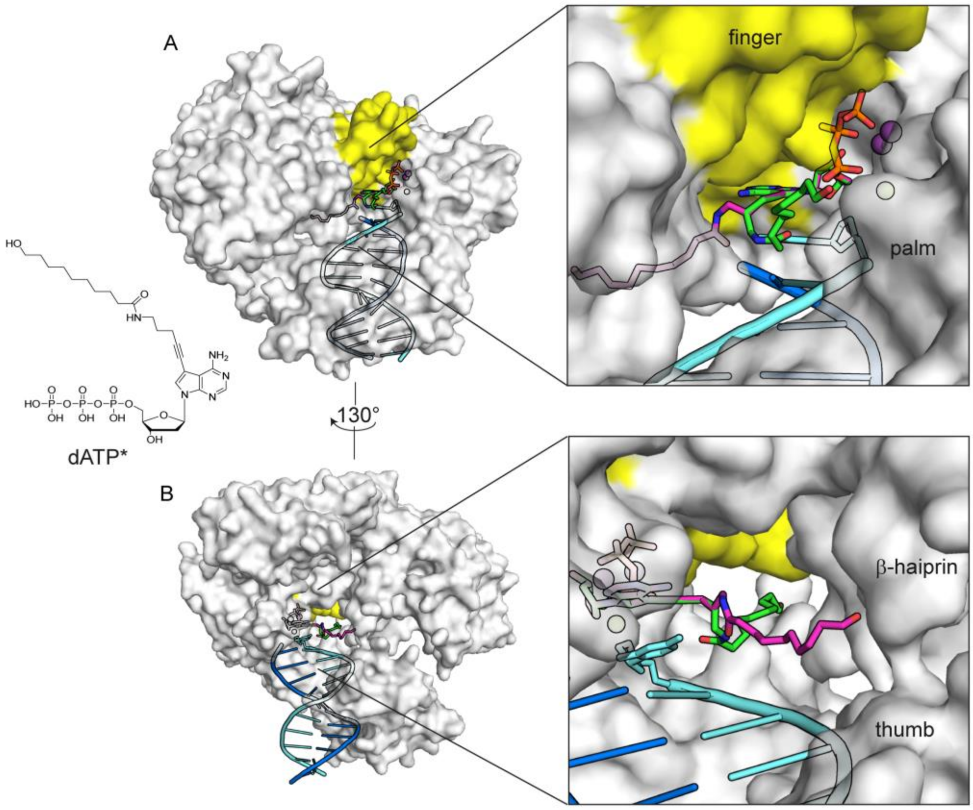

S10 Fig. Modelling of a modified nucleotide into KOD DNA pol. The 7-(N-(10-hydroxydecanoyl)-aminopentinyl)-7-deaza-2-dATP (dATP*) (PDB Code: 0L3) was modelled into the active site of KOD DNA pol. The nucleotide moiety of the dATP* was superimposed with the dATP of the KOD DNA pol structure and the linker was modelled in two conformations (green and pink sticks) into the free space within the enzyme using COOT [1]. (A) View onto the Hoogsteen side of the dATP* showing the linker modelled in green pointing towards the finger and palm domain. (B) Rotation of approx. 130° showing the linker modelled in pink pointing towards the thumb domain and the β-hairpin.

1. Emsley P, Lohkamp B, Scott WG, Cowtan K. Features and development of Coot. Acta Crystallogr, Sect D: Biol Crystallogr. 2010;66(4):486-501. doi: doi:10.1107/S0907444910007493.
